# Diversity and Composition of the Microbiome Associated with Adult of the Green Shield Bug *Palomena prasina* (Hemiptera: Pentatomidae)

**DOI:** 10.1007/s00248-026-02779-2

**Published:** 2026-04-29

**Authors:** Naciye Sena Çağatay, Aslı Dageri, Islam Saruhan, Celal Tuncer, Nurper Guz

**Affiliations:** 1 Department of Plant Protection, Molecular Entomology Laboratory, Faculty of Agriculture, University of Ankara, Ankara, Türkiye; 2 Institute of Infection, Veterinary and Ecological Sciences, University of Liverpool, Liverpool, UK; 3 Department of Plant Protection, Agricultural Faculty, Acarology Laboratory, Cukurova University, 01330 Adana, Türkiye; 4 Department of Molecular Biology and Genetics, Faculty of Science, Necmettin Erbakan University, 42090, Konya, Meram, Türkiye; 5 Department of Plant Protection, Faculty of Agriculture, Ondokuz Mayıs University, Samsun, Türkiye; 6 Biotechnology Institute, Ankara University, Gümüşdere Yerleşkesi Keçiören, Ankara, Türkiye

**Keywords:** Symbionts, *Pantoea*, *Sodalis*, Metagenomic, Insect-bacteria interaction

## Abstract

Hazelnut is a major export commodity for Türkiye, the world’s leading producer, yet pest pressure in hazelnut orchards has caused substantial quantitative and qualitative yield losses in recent years. Among emerging pests, the green shield bug (GSB) *Palomena prasina* (Hemiptera: Pentatomidae) has become a key threat due to direct feeding on developing fruits. Despite its increasing economic relevance, the microbial community associated with *P. prasina* remains poorly characterized. Here, we present the first comprehensive analysis of the bacterial community associated with *P. prasina* using 16 S rRNA gene metabarcoding combined with prevalence screening and phylogenetic analyses. A total of 36 bacterial taxa were detected across sampled populations, with *Pantoea* and *Sodalis* identified as the dominant genera. Bacterial diversity did not differ significantly between sexes or among geographic locations, indicating a relatively stable microbial community. Prevalence analyses revealed that *Pantoea* spp. were present in all examined individuals, whereas *Sodalis* spp. showed variable infection frequencies among populations. Phylogenetic reconstruction indicated contrasting evolutionary patterns between these dominant taxa, with *Pantoea* lineages displaying a polyphyletic structure suggestive of repeated environmental acquisition, while *Sodalis* sequences formed a more cohesive, host-associated lineage consistent with a facultative symbiotic lifestyle. Overall, these findings improve our understanding of stink bug-microbe associations and provide an ecological framework for future studies exploring symbiont-based pest management strategies.

**Supplementary Information:**

The online version contains supplementary material available at 10.1007/s00248-026-02779-2.

## Introduction

Türkiye is the world’s leading producer of hazelnut (*Corylus avellana* L.), contributing approximately 62% of global output and cultivating around 665,000 tons of shelled hazelnuts annually across 735,000 hectares [1]. Additionally, Türkiye meets nearly 75% of global hazelnut demand, making the crop economically critical both nationally and internationally. Hazelnut export revenues constitute a major component of Türkiye’s agricultural economy and support the livelihoods of nearly 500,000 farming families [1, 2]. However, production stability has become increasingly threatened by rising pest pressure, which has intensified across many hazelnut-growing regions. In addition, shifting ecological conditions make the situation even more complex and highlight the need to better understand the biological factors that drive pest outbreaks. According to 2019 statistics from the Turkish Statistical Institute [3], hazelnut production in Türkiye is concentrated primarily in Ordu (30.5%), followed by Samsun (16.2%), Giresun (15.8%), Sakarya (10.6%), Trabzon (8.8%), and Düzce (8.5%). Although hazelnut cultivation areas fluctuate across years and regions, numerous sap-sucking insect species continue to threaten both yield and nut quality. Among these pests, the hazelnut weevil (*Curculio nucum* L., Coleoptera: Curculionidae), and the green shield bug, (*Palomena prasina* L., Hemiptera: Pentatomidae) are considered the two most economically damaging species [4–6]. A previous survey reported that among piercing-sucking insects in Turkish hazelnut orchards, *P. prasina* was the dominant species, representing approximately 85% of individuals collected [6]. In recent years, changes in orchard management alongside reduced natural enemy pressure may have facilitated the continued rise of *P. prasina* as a major pest.

Symbiotic associations between insects and microorganisms such as bacteria, viruses, and fungi have been well documented. These partnerships, which involve the close coexistence of different organisms, can range from parasitic to highly beneficial interactions [7, 8]. Beneficial bacterial symbionts play key roles in nutrient supplementation, stress tolerance, detoxification, and defense against natural enemies [9–11]. They also influence ecological dynamics by shaping host physiology and interactions with pathogens and natural enemies [12]. Insects typically harbor both primary (obligate) and secondary (facultative) symbionts, which differ in their essentiality, localization, and modes of transmission [13, 14].

In stink bugs (Hemiptera: Pentatomidae), gut-associated Gammaproteobacteria are key components of the host microbiome. Obligate symbionts, most commonly *Pantoea*-like [15, 16] or *Erwinia*-like bacteria [17, 18], are typically housed in specialized crypts of the posterior midgut, where they contribute to host development, nutrient provisioning, and overall fitness [15, 16, 18]. In contrast, facultative symbionts such as *Sodalis* spp [19–21]. may occur at lower prevalence and colonize a wider range of tissues, including gut regions outside the crypts or other organs. The loss or disruption of obligate symbionts can severely affect host performance, resulting in delayed growth, increased mortality, and reduced fitness [16, 18]. In pentatomid insects, these symbionts are vertically transmitted via egg smearing, whereby females deposit symbiont-containing secretions on the egg surface during oviposition, enabling acquisition by newly hatched nymphs [22–25]. Although symbiotic associations have been extensively studied in sever al stink bug species, the microbial community of *Palomena prasina* remains relatively poorly characterized. Although gut-associated symbionts have been localized in the V4 region of the *P. prasina* midgut [25], knowledge of the overall diversity, stability, and functional roles of the associated bacterial community remains limited. This knowledge gap is particularly relevant given the emergence of *P. prasina* as a major hazelnut pest and the growing interest in symbiont-based control strategies [22–26].

In general, pest management relies heavily on intensive chemical pesticide use, which poses significant risks to environmental and human health and promotes the rapid evolution of resistance in pest populations. These limitations have stimulated increasing interest in alternative strategies, including the manipulation of insect-associated symbionts as tools for integrated pest management. The control of *P. prasina* is further complicated by the scarcity of effective natural enemies and limited knowledge of its ecological dynamics across different agroecosystems. Moreover, widespread pesticide application in Türkiye and worldwide reduces the efficacy of biological control agents, underscoring the need for sustainable, microbe-based approaches. In recent years, symbiotic bacteria have attracted growing attention as targets or tools for controlling agricultural and public health pests, and numerous studies have investigated their diversity and functional roles in Hemiptera [18–28]. In this study, specimens of *P. prasina* were collected from major hazelnut-producing provinces in Türkiye, and their bacterial symbionts were characterized using a 16 S rRNA metagenomic approach. The study also examined the prevalence of the two most abundant bacterial species detected *Pantoea* spp. and *Sodalis* spp. within *P. prasina* populations. In addition, molecular phylogenetic relationships were analysed using 16 S rRNA sequences belonging to the genera *Pantoea* and *Sodalis*. Specifically, we addressed the following questions: (i) What is the symbiotic bacterial composition of *P. prasina*? (ii) Do these communities vary by geography or sex? (iii) What are the phylogenetic relationships of the dominant symbionts? By addressing these questions, this study provides essential knowledge for future symbiont-based interventions in hazelnut agroecosystems. It also presents the first metagenomic characterization of the symbiotic bacterial community of *P. prasina*. Furthermore, by integrating prevalence and phylogenetic placement of its dominant symbionts, the study offers a novel and foundational contribution to the species symbiont biology.

## Materials and Methods

Adult individuals of *Palomena prasina* were collected from hazelnut orchards located in major hazelnut-producing provinces of Türkiye. Two sampling schemes were used in this study. For the metagenome analysis, a total of 90 adult individuals were collected from five provinces, with three districts sampled per province and six individuals (three females and three males) collected from each district. From these samples, 30 individuals were selected for 16 S rRNA gene-based metagenome sequencing to characterize the bacterial community associated with *P. prasina*. Based on the metagenome results, *Sodalis* and *Pantoea* were identified as dominant bacterial taxa. Therefore, an additional sampling was conducted to determine the prevalence of these symbiotic bacteria in field populations. For this purpose, 600 adult individuals were collected, with 40 individuals sampled from each district (120 individuals per province). Prior to DNA extraction, specimens were surface sterilized to remove external microorganisms, and DNA extraction was performed using the same protocol for all experiments. Detailed descriptions of the sampling design and laboratory procedures are provided in the following sections.

### *Palomena prasina* 16 S rRNA Metagenome Analysis

#### Sample Collection and Identification

**Fig. 1:**
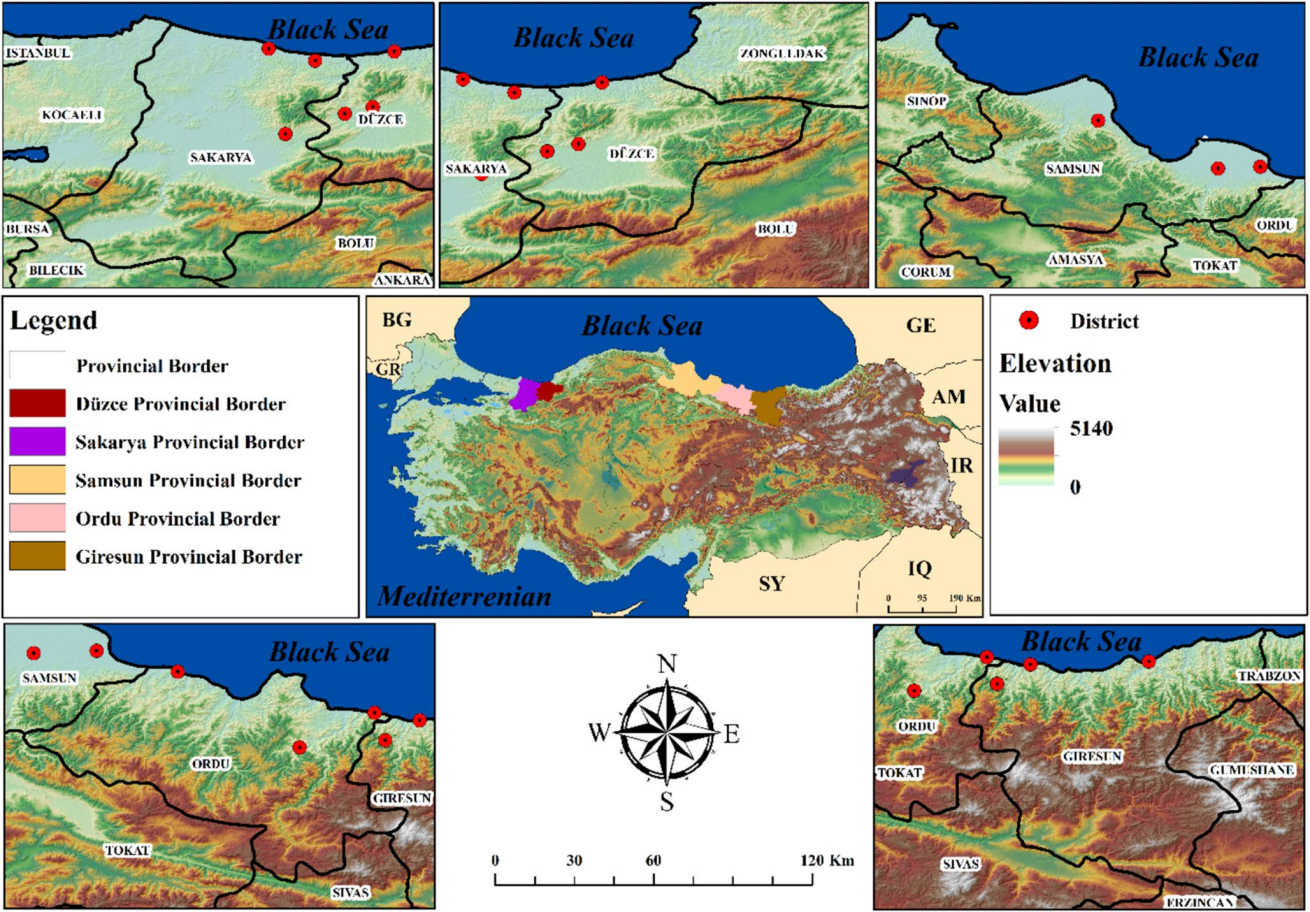
Map showing the sampling locations of *Palomena prasina* populations collected in Türkiye. Red circles indicate the sites where specimens were collected

Adult individuals of *Palomena prasina* were collected from hazelnut orchards located in provinces that together account for approximately 80% of Türkiye’s hazelnut production (Ordu, Giresun, Samsun, Sakarya, and Düzce) using the beating-sheet method in early May (Fig. 1). Sampling was conducted by selecting three districts from each province and three orchards from each district (Supplementary Table 1). Species identification was performed according to morphological characteristics described by [29, 30].

Before preservation, the ventral side of the abdomen of each adult was examined to determine sex. Gender discrimination was carried out by examining the external genitalia, and each individual was recorded as male or female and stored in cryovial tubes (Supplementary Fig. 1).

#### DNA Extraction

The metagenome analysis, one male and one female with high-quality DNA were selected from each district, resulting in 30 individuals. Sex-specific pools were created to ensure representation across districts and to allow comparisons between sexes.

Prior to DNA extraction, each specimen was surface-sterilized by washing with 1% NaOCl for 1 min, followed by rinsing with distilled water for 1 min, repeated twice. Samples were then air-dried on sterile paper. Total genomic DNA was extracted from each specimen using the High Pure DNA Isolation Kit (Roche, USA) following the manufacturer’s instructions.

DNA quality was evaluated using a NanoDrop 2000 spectrophotometer (Thermo Scientific, USA) and by electrophoresis on a 1% agarose gel stained with PronaSafe (Laboratorios Conda, S.A.).

#### 16 S rRNA Gene Amplicon Sequencing

Genomic DNA was quantified prior to high-throughput sequencing on the Illumina platform (Illumina, San Diego, CA, USA). The V3–V4 region of the 16 S rRNA gene was amplified using primers 341 F and 805R, which included Illumina adapter sequences [31]. Sequencing was performed on an Illumina MiSeq platform, generating 2 × 150 bp paired end reads.

#### Bioinformatic Analyses

Raw sequences were processed using QIIME2 (v2018.8.0) [32]. Adapter sequences were removed and reads were quality filtered (Q > 20, N bases < 1%) [33]. Paired-end reads were merged with FLASH (v1.2.11) [34]. Chimeric sequences were identified and removed with USEARCH (v7.0.1090) using UCHIME (v4.2.40) [35], and operational taxonomic units (OTUs) were clustered at 97% identity with the UPARSE-OTU algorithm [36]. Representative sequences were taxonomically classified with the RDP Classifier (v2.2) trained on the Greengenes database v201305. Venn diagrams showing shared OTUs between genders and among locations were generated in R (v3.1.1) using the Venn Diagram package [37]. Alpha diversity metrics, including ACE [38], Chao1 [39], Shannon, and Simpson [40] were calculated in QIIME2 and RStudio (v4.2.0). Paired-end reads were generated using Illumina MiSeq platform, and the data processing outputs are summarized in Supplementary Table 5. Each individual sample contained an average of 137,588 reads, with an average read length of 252 bp. A total of 4,127,665 V3-V4 16 S rRNA sequences were obtained from all samples. After quality filtering, sequences were clustered into operational taxonomic units (OTUs) at 97% similarity, and the detailed clustering statistics are provided in Supplementary Table 5.

#### Statistical Analyses

Alpha diversity differences between genders were tested with the Wilcoxon rank-sum test [41]. Differences in relative genus abundances across sampling locations were evaluated using the Kruskal-Wallis test [42].

### Determination of the Prevalence *Pantoea* spp. and *Sodalis* spp. in Field Samples

#### Sample Collection and DNA Extraction

DNA extraction was performed as described in Sect. 2.1.2. For the prevalence analysis, a total of 600 adult individuals were collected. In each district, one representative hazelnut orchard was selected, and two individuals were randomly sampled from each of 20 sampling points within the orchard. To verify the presence of *P. prasina* DNA in the extracted samples, species-specific primers (Supplementary Table 2) were used to amplify insect DNA from a total of 600 adult specimens. DNA templates passing this quality control (QC) stage were then used for onward detection of symbionts through PCR assays. Each PCR assay was performed in a 50 µL reaction volume, containing 5 µL GoTaq 5xPCR Buffer (Promega, USA), 1 µL each forward and reverse primer (concentration 10 pmol/µL), 1 µL dNTPs mix (10 mM, Thermoscientific, USA), 0,5 µl Taq DNA polymerase (5 U/ul, Promega, USA), ~ 30.5 µL nuclease-free water and ~ 1 µL Template DNA (200ng). All PCR assays were performed under the following thermal conditions: initial denaturation at 94 °C for 3 min, followed by 35 cycles of denaturation (94 °C for 1 min), annealing (45 °C for 1 min), extension (72 °C for 2 min), and with a final extension at 72 °C for 10 min. Each PCR run included positive controls (confirmed *P. prasina* DNA) and negative controls (no-template controls).

#### Screening and Sequencing of Bacteria Using PCR

Based on the metagenomic results, species-specific primers targeting *Pantoea* and *Sodalis* were designed by using Primer3 software (Supplementary Table 2). Endpoint PCR targeting the 16 S rRNA gene were performed to screen for the presence of these bacteria. Each PCR reaction was carried out in a 50 µL reaction volume with the following thermal cycling conditions: initial denaturation at 94 °C for 3 min, followed by 35 cycles of denaturation at 94 °C for 1 min, annealing at Ta for 1 min, and extension at 72 °C for 2 min, with a final extension at 72 °C for 10 mix. The annealing temperature varied by primer pair, and detailed primer information including sequences, annealing temperatures, and expected amplicon sizes is provided in Supplementary Table 2. Each PCR run included positive controls (individuals known to be infected with target bacteria) and negative controls (no-template controls).

PCR products were visualised on 1.5% agarose gels stained with Pronosafe Nucleic Acid Staining Solution (Laboratorios, Conda, S.A.). Based on the gel results, each individual *P. prasina* was assigned a symbiont infection status (presence/absence) for both *Pantoea* and *Sodalis.* PCR products were purified using a GeneAll Gel Extraction Kit (GeneAll Biotechnology, Seoul, Korea). Sequencing reactions were performed using the DTCS Quick Start Kit (Beckman Coulter), followed by ethanol-based purification, as recommended in the DTCS Quick Start Kit protocol. Purified sequencing reactions were analysed on the GenomeLab GeXP Genetic Analysis System (GeXP CEQ 8800; Beckman Coulter, CA, USA).

#### Statistical Analysis

The abundance of the genus *Sodalis* in *P. prasina* specimens collected from different regions was quantified using the following formula:

$$\:\mathrm{Infection}\text{ rate }{(\%)}=\frac{\text{Number of infected individuals}}{\text{Total number of individuals}}\times\:100$$

The data were analyzed using Pearson’s Chi-square tests to assess whether the association between infection status and population (at the province and district levels) or sex was statistically significant [43]. Differences in *Sodalis* infection rates among provinces were further evaluated using a one-way ANOVA, followed by Tukey’s HSD post-hoc test to identify pairwise differences [44]. Statistical significance was set at *P* < 0.05. All analyses were performed in R software (R Core Team, 2020).

### Phylogenetic Analysis

The *Sodalis 16 S rRNA* sequences (Supplementary Table 3) and the *Pantoea 16 S rRNA* dataset (Supplementary Table 4) were aligned with MAFFTv7.4 [45]. BMGE version 1.12 [46] was employed to trim ambiguously aligned regions from the alignments. The *Sodalis* dataset consist of 111 sequences (Supplementary Table 3) and *Pantoea* dataset consisted of 102 sequences, all retrieved from Genbank (Supplementary Table 4). Modelfinder [47] was used to determine the best-fitting substitution model. Phylogenetic relationships were inferred using IQTREE [48] with 1000 rapid bootstrap replicates [49].

The dataset for the genus *Pantoea* is composed of 102 nucleotide sequences. ModelFinder analysis, performed using IQ-TREE version 1.6.12, identified K2P + I+G4 as the best-fitting model based on the Bayesian Information Criterion (BIC). The K2P model, or Kimura 2 parameter model [49], evaluates differences in transition and transversion rates as well as substitution rates among sites. The “I” denotes invariant sites, while G4 (Gamma with four categories) models rate heterogeneity across sites.

## Results

### Microbiome Composition Based on Illumina Amplicon Sequencing

Across all analyses, *Pantoea* and *Sodalis* clearly emerged as the dominant and most consistent detected symbionts associated with *P. prasina* populations. The OTU table generated from 16 S rRNA gene sequencing of the V3–V4 region using the Illumina MiSeq platform is provided in Supplementary Table 5.

**Fig. 2:**
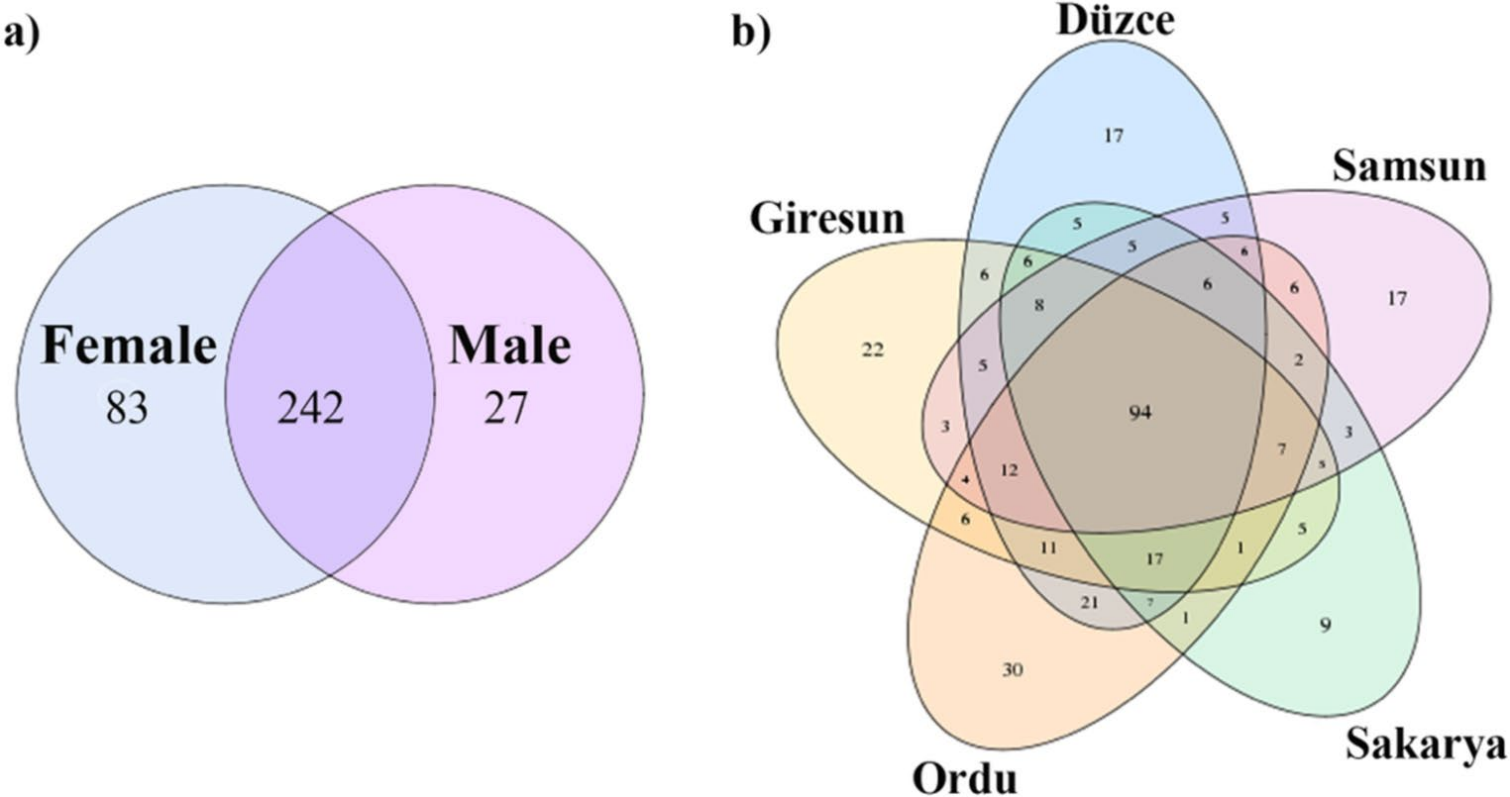
Venn diagrams showing the number of shared and unique operational taxonomic units (OTUs) among *Palomena prasina* samples according to (**a**) gender and (**b**) sampling locations

**Fig. 3:**
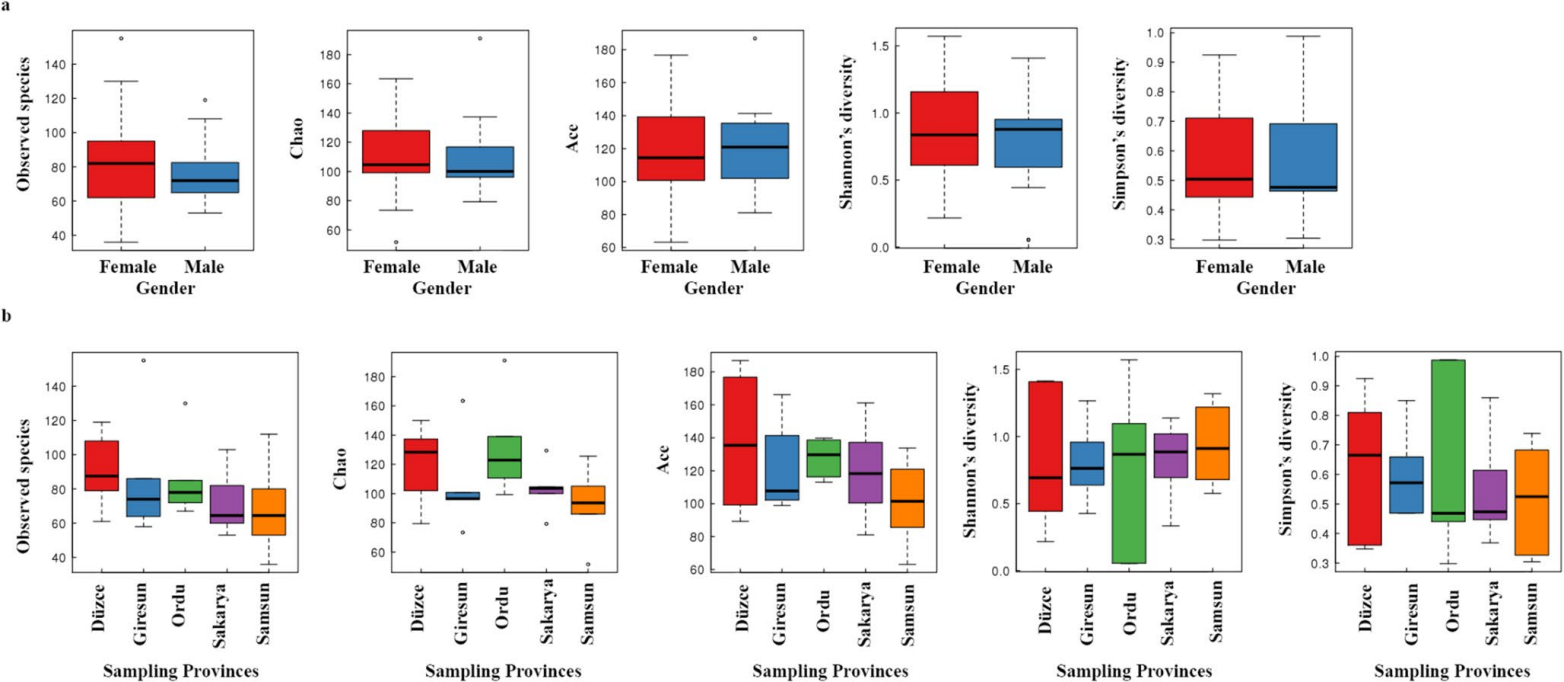
Boxplots showing the alpha diversity of bacterial communities associated with *Palomena prasina* samples based on (**a**) gender and (**b**) sampling provinces

**Fig. 4:**
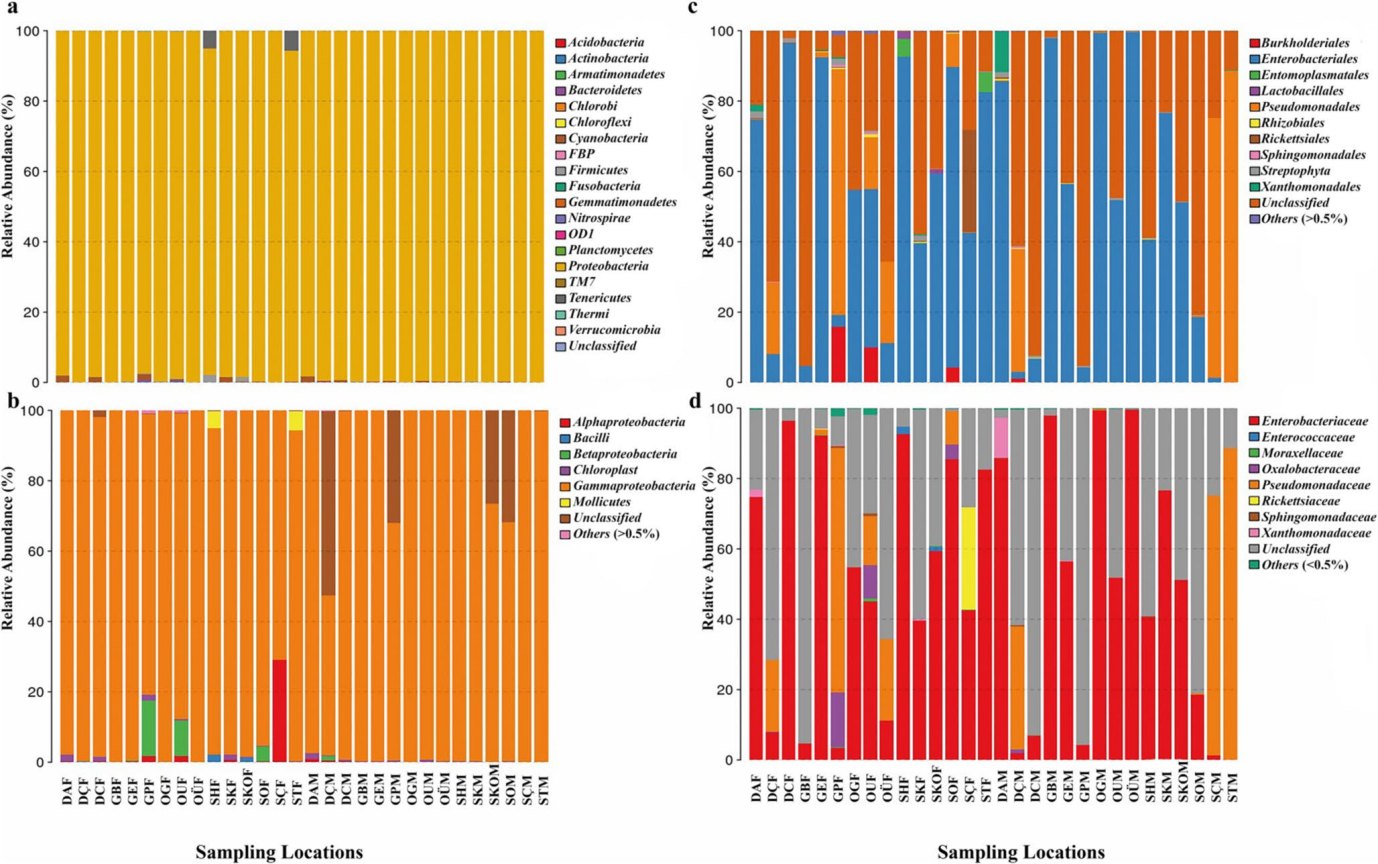
Stacked bar plots showing the relative abundance of bacterial taxa associated with Palomena prasina samples at different taxonomic levels: (**a**) phylum, (**b**) class, (**c**) order, and (**d**) family. Sample codes correspond to location and sex (see Sample ID; Supplementary Table 5 )

Comparison of OTU richness between female and male individuals revealed 83 OTUs unique to females and 27 unique to males, while 242 OTUs were shared between the two sexes. At the provincial level, the number of unique OTUs detected was 30 in Ordu, 22 in Giresun, 17 in Samsun, and 9 in Sakarya, whereas Düzce also exhibited province-specific OTUs. In total, 94 OTUs were shared across all sampling locations (Fig. 2). Although minor variation was observed in overall bacterial diversity, alpha diversity indices (Chao, ACE, Shannon, Simpson) showed no statistically significant differences (*P* > 0.05) between genders or among provinces (Fig. 3). The bacterial community associated with *P. prasina* was dominated by the phyla Proteobacteria, Cyanobacteria, and Firmicutes, with Proteobacteria representing the most abundant group. Within this phylum, Gammaproteobacteria was the dominant class, and the orders Enterobacterales and Pseudomonadales were frequently detected. The families Enterobacteriaceae and Pseudomonadaceae were commonly observed, with *Pantoea* and *Sodalis* representing the predominant genera across sampling sites (Fig. 4; Supplementary Fig. 2).

In addition, an unidentified OTU belonging to the order *Burkholderiales* was detected at relatively high abundance in several samples. However, due to the limited taxonomic resolution of 16 S rRNA-based metabarcoding and the lack of additional supporting data, its ecological role in *P. prasina* could not be determined.

### Prevalence of Most Common Symbiotic Bacteria (*Pantoea* and *Sodalis*) Across Different Districts from Türkiye

The distribution and infection rates of *Pantoea* and *Sodalis* across *Palomena prasina* populations from different provinces are provided in Supplementary Table 6. *Pantoea* infection was detected in 100% of the 600 individuals screened, strongly supporting its role as an obligate symbiont. *Sodalis* spp. were detected in 409 out of 600 samples, corresponding to an overall prevalence of 68.2%. Additionally, fifteen randomly selected positive samples from each location were sequenced to further validate the PCR-based detection and to confirm the specificity of the species-specific primers for the target bacteria. BLASTn analysis of the obtained sequences showed high similarity to reference sequences deposited in the NCBI database. The sequences exhibited 100% and 99.26% nucleotide identity, with query coverage of 100% and E-values of 0.0, to *Pantoea ananatis* (CP054912.1, MT367861.1), *Pantoea agglomerans* (MT367841.1), and *Sodalis* endosymbionts (MF429882.1, AB915782.1), confirming the molecular identification of these bacteria.

Data on *Sodalis* infection in *P. prasina* were analyzed using Pearson’s Chi-square tests to evaluate differences in infection prevalence between sexes, provinces, and districts. The analyses revealed no significant difference between sexes (χ² = 2.23, df = 1, *P* = 0.136). In contrast, significant differences were detected among provinces (χ² = 50.57, df = 4, *P* < 0.001) as well as among districts within provinces (χ² = 85.13, df = 14, *P* < 0.001).

**Fig. 5:**
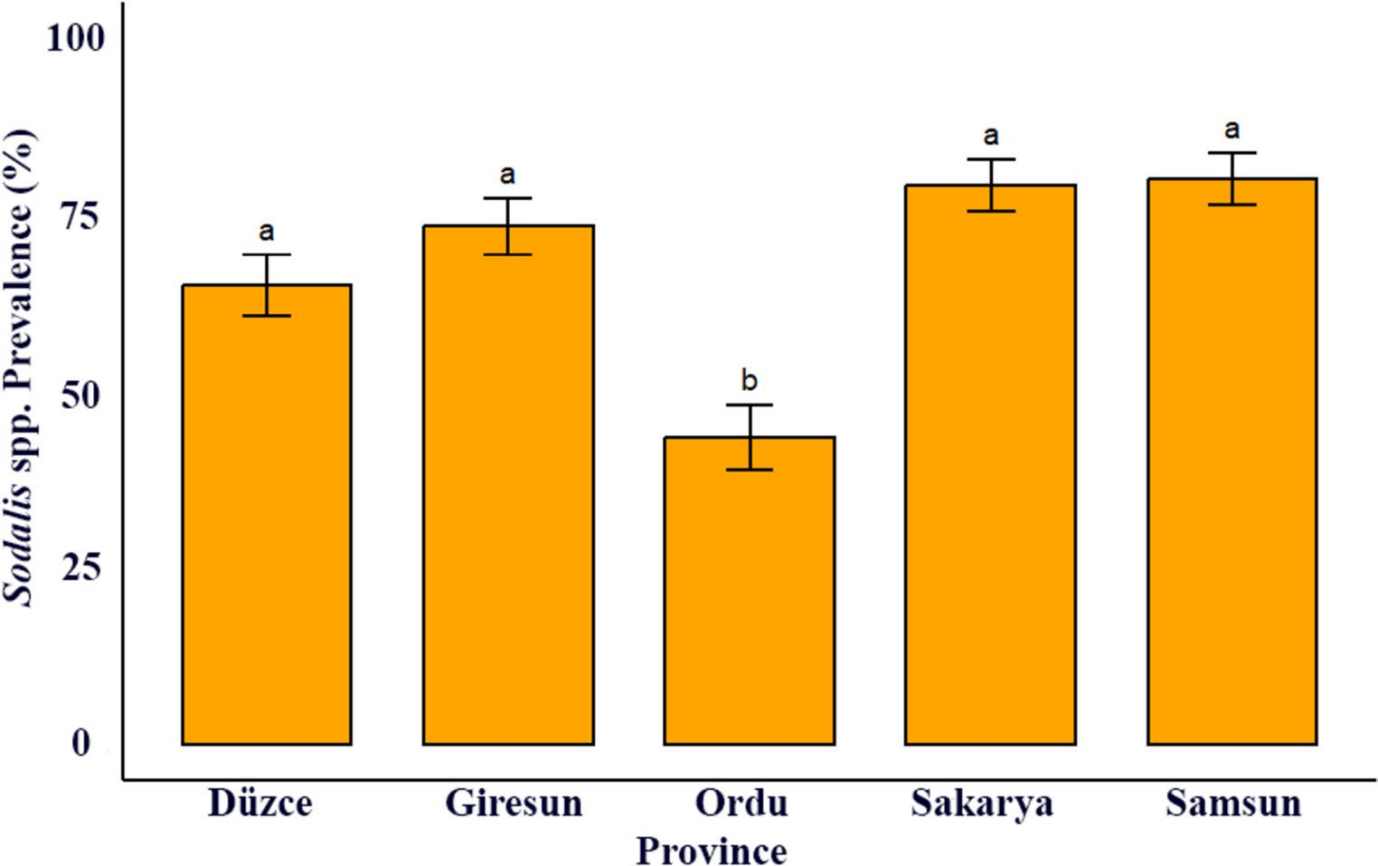
*Sodalis* infection rates (± SE) across sampling provinces (*n* = 120 per province). Different letters indicate significant differences among provinces according to Tukey’s HSD test (*P* < 0.05)

Because infection rates differed significantly among provinces, a one-way ANOVA was conducted to compare mean infection levels across provinces. The analysis revealed a highly significant difference in mean infection rates among provinces (F_4, 595_ = 13.69, *P* < 0.001). Following the significant ANOVA, Tukey’s HSD post-hoc test was performed to identify pairwise differences. The test indicated that Ordu had significantly lower infection rates compared to Düzce, Sakarya, and Samsun (*P* < 0.05), whereas differences among the remaining provinces were not statistically significant (Fig. 5).

These results suggest that province is a key factor influencing *Sodalis* infection rates in *P. prasina*, while sex does not appear to have a significant effect.

### Phylogenetic Tree

**Fig. 6:**
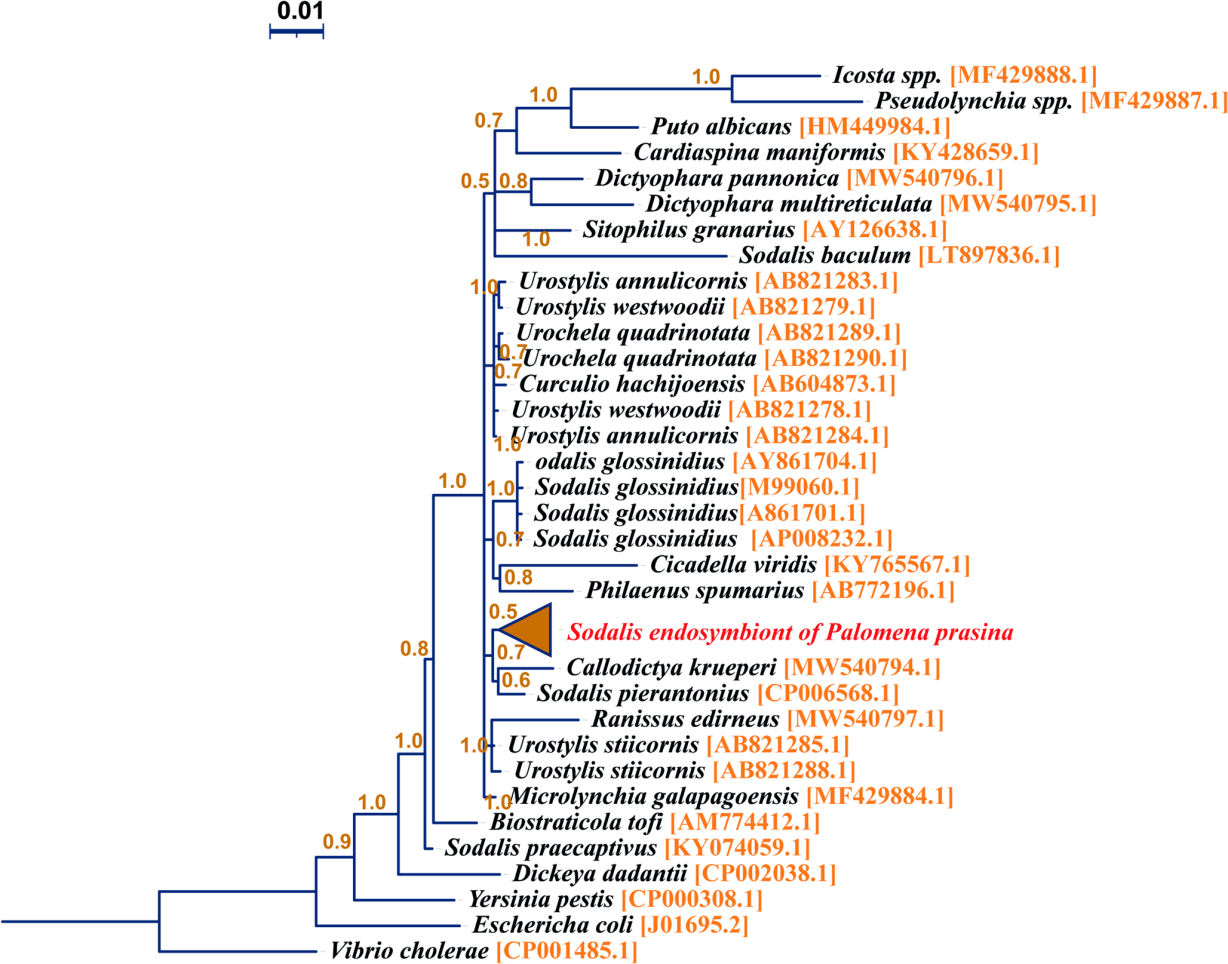
Bayesian phylogenetic tree showing the phylogenetic relationships among *Sodalis* species based on 16 S rRNA gene sequences. Sequences obtained in this study are highlighted in red. Posterior probability values are indicated at the nodes. The scale bar represents the number of substitutions per site

A Bayesian phylogenetic tree was generated from a *Sodalis* genus dataset consisting of 111 nucleotide sequences. The analysis was performed using MrBayes v3.2.7 software with the GTR substitution model (Fig. 6). The resulting phylogenetic tree revealed that the *Sodalis* genus is divided into six well-supported clusters. The sequences identified from *P. prasina* formed a distinct, well-supported clade with high posterior probability, indicating clear phylogenetic separation from *Sodalis* strains associated with other hosts.

**Fig. 7:**
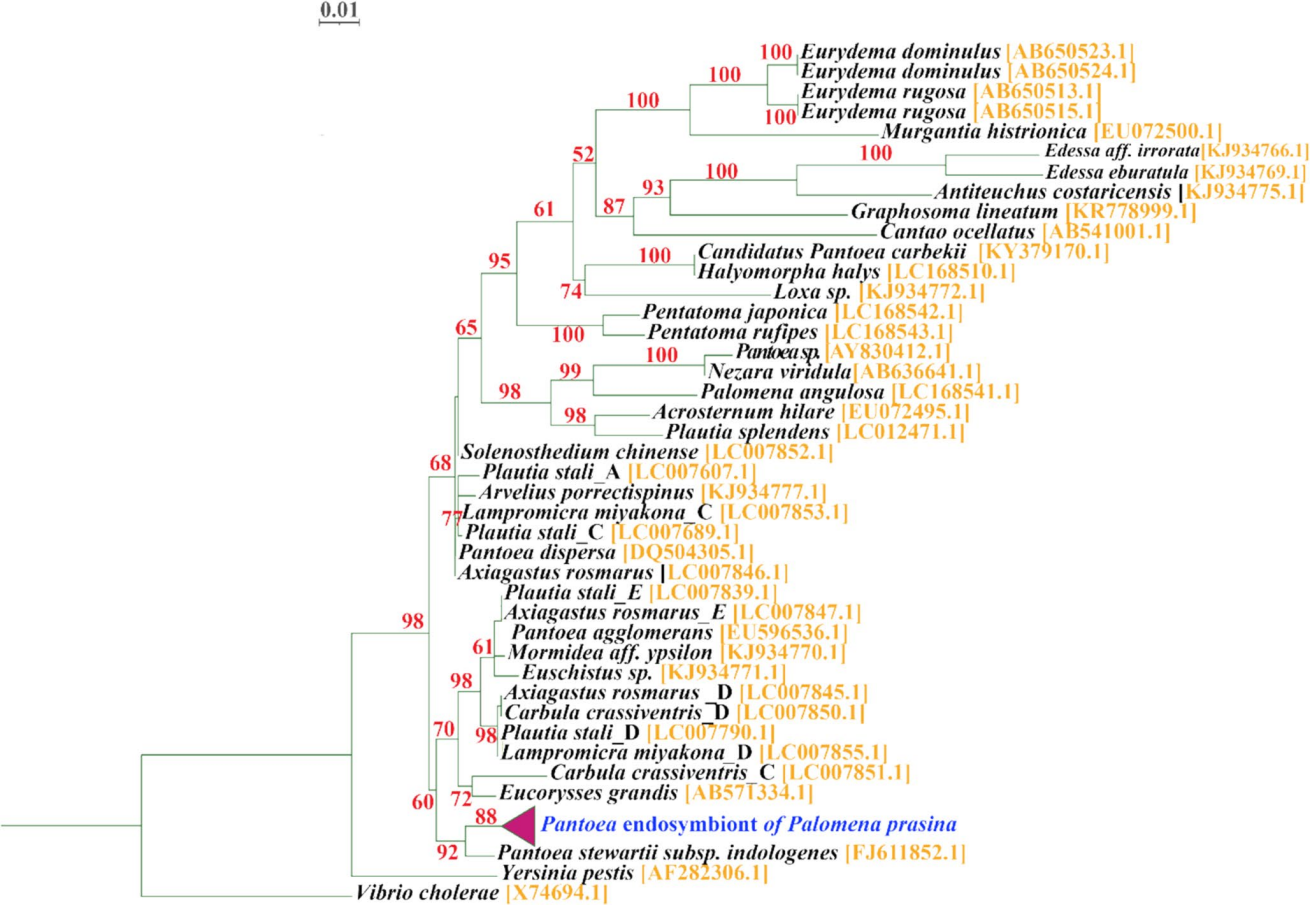
Maximum likelihood phylogenetic tree showing the phylogenetic relationships among *Pantoea* species based on 16 S rRNA gene sequences. Sequences obtained in this study are highlighted in blue. Bootstrap values are indicated at the nodes. The scale bar represents the number of substitutions per site

The resulting maximum likelihood phylogenetic tree is presented in Fig. 7. The *Pantoea* sequence obtained from *P. prasina* formed a well-supported clade within Enterobacteriaceae, clustering most closely with *Pantoea ananatis* and *Pantoea agglomerans*, in agreement with BLAST-based taxonomic assignments. Phylogenetic analysis of *Pantoea* strains isolated from *P. prasina* and related species revealed two principal clades. However, branch lengths suggest that the *P. prasina* associated sequence is phylogenetically distinct rather than representing a strain of *P. stewartii*, although it shows broader evolutionary affinity within this lineage.

## Discussion

Effective management of hazelnut pests increasingly requires sustainable alternatives to conventional insecticides. As one of the major pests in hazelnut orchards, *P. prasina* highlights the need for novel approaches to identify effective control strategies that do not rely solely on chemical inputs. In this context, insect-associated symbiotic bacteria have gained increasing attention. The variation in *Sodalis* prevalence among provinces may be associated with environmental differences and the biological roles of symbiotic bacteria in Hemiptera, which can affect host development, nutrition, and ecological adaptation. Previous studies have shown that symbiotic bacteria in Hemiptera can influence host nutrition, development, and ecological performance, and their prevalence may vary depending on local environmental conditions and host-symbiont interactions. Understanding the microbiome of *P. prasina* is therefore a critical first step toward evaluating its potential for symbiont-based management strategies [22]. Symbiont-based approaches may include disrupting essential bacterial partners, such as *Pantoea*, to impair host development or reproduction. Additionally, paratransgenic strategies represent a promising biotechnology avenue. In these approaches, engineered symbionts are used to deliver molecules that reduce pest survival. Microbiome manipulation, through selective introduction or removal of symbionts, could also alter physiological pathways critical for host performance [23–25]. As the obligate functions of *Pantoea* and the facultative contributions of *Sodalis* become clearer, the potential of symbiont-targeted strategies will become more apparent. These approaches may offer environmentally safe, species-specific, and sustainable alternatives to conventional chemical insecticides. However, any symbiont-targeted intervention must carefully consider potential ecological side-effects, particularly because *Pantoea* species are widely distributed in agricultural environments, where they can act as plant endophytes, epiphytes, plant growth-promoting bacteria, or opportunistic pathogens [50–52]. Understanding these ecological roles is essential to ensure that disrupting *Pantoea* in *P. prasina* does not inadvertently impact non-target plants or soil microbial communities.

In this study, we characterized the microbiome of *P. prasina* collected from major hazelnut producing provinces in Türkiye using a 16 S rRNA-based metagenomic approach. Bacterial diversity did not differ significantly between males and females or among provinces. The absence of sex or region-specific differences suggests that individuals experience similar dietary and ecological conditions across the sampled landscape. This uniformity may reflect the relatively homogeneous microhabitat structure of hazelnut orchards [53]. Long-term feeding predominantly on hazelnut in these regions may have contributed to the observed stability of the microbiome. Such stability may represent a species-specific feature of *P. prasina* and is consistent with evidence that intestinal symbiotic bacteria can remain relatively stable at the species level in Pentatomidae [54, 55]. Targeted experimental studies will be required to identify the ecological and physiological factors that maintain this stable microbiome structure.

The OTU analysis also reflects this general stability. While some province-specific and sex-specific OTUs were detected, these represented a small fraction of the total diversity, and most OTUs were shared broadly among individuals. The finding that all provinces shared a common set of 94 OTUs indicates that *P. prasina* maintains a highly conserved core bacterial community despite environmental variation across sampling sites. Such patterns suggest that rare or locally restricted OTUs likely represent transient environmental bacteria. In contrast, the dominant community appears to be shaped primarily by the species consistent feeding habits and the relatively homogeneous structure of its habitat.

Our metagenomic analysis revealed a diverse bacterial community dominated by Proteobacteria, particularly Gammaproteobacteria within the Enterobacteriaceae. Although many arthropods commonly harbour Alphaproteobacteria [56], our data show that *P. prasina* is instead dominated by Gammaproteobacteria, consistent with previous findings in Pentatomidae and other Hemiptera [18, 21, 22, 28].This taxonomic pattern suggests that Gammaproteobacteria play a central role in the gut ecology of sap sucking bugs and may contribute to the ecological success of *P. prasina* in hazelnut orchards.

Metagenomic analysis of the collected GSB specimens revealed the presence of 36 bacterial genera, including *Pantoea*, *Spiroplasma*, *Sodalis*, *Escherichia/Shigella*, *Anaplasma*, *Enterococcus*, *Erwinia*, *Streptophyta*, *Rhizobium*, *Gibbsiella*, *Arsenophonus*, *Cronobacter*, *Sphingomonas*, *Xanthomonas*, *Propionibacterium*, *Citrobacter*, *Luteibacter*, *Prevotella*, *Acinetobacter*, *Plesiomonas*, *Klebsiella*, *Williamsia*, *Lelliotia*, *Micrococcus*, *Flavisolibacter*, *Paenibacillus*, *Rickettsia*, *Massilia*, *Candidatus Brocadia*, *Armatimonadetes gp5*, *Raoultella*, *Pseudomonas*, *Stenotrophomonas*, *Serratia*, *Enterobacter*, and *Azotobacter*. Among these, *Pantoea*, *Raoultella*, *Pseudomonas*, *Stenotrophomonas*, and *Serratia* have previously been reported in GSB [25, 57]. To our knowledge, the remaining 31 genera are reported here for the first time from GSB.

Interestingly, no *Wolbachia* were detected, despite its presence being documented in several Hemiptera species [58–60]. The absence of *Wolbachia* is notable given its widespread distribution across diverse Hemiptera lineages, where it often influences host reproduction, immunity, thermal tolerance, or pathogen susceptibility. Its complete lack in all populations of *P. prasina* may indicate that this species does not rely on *Wolbachia*-mediated reproductive manipulation or fitness enhancement, unlike many other heteropterans [58, 59]. Several ecological explanations may account for this pattern. The stability of the species vertically transmitted gut-symbiont system may reduce opportunities for *Wolbachia* acquisition, and strong competitive exclusion by dominant gut bacteria such as *Pantoea* and *Sodalis* could further limit its establishment. Alternatively, local environmental or climatic factors in hazelnut orchards may not favour the persistence or transmission of *Wolbachia*. Further screening across broader geographic ranges and developmental stages will be essential to determine whether *Wolbachia* is completely absent in this species or occurs at low, undetectable frequencies. In several stink bug species, *Burkholderia*-related bacteria are known to form beneficial associations with their hosts [61, 62]. However, due to the limited taxonomic resolution of 16 S rRNA-based metabarcoding and the lack of additional supporting data, the ecological role of this OTU in *P. prasina* remains unclear.

Metagenomic analyses revealed that *Pantoea* was one of the most abundant genera associated with *P. prasina*, a pattern that was further supported by PCR-based detection of *Pantoea* in all 600 examined individuals. Similar associations between Enterobacteriaceae, particularly *Pantoea*, and Pentatomidae have been widely reported, indicating that members of this genus are recurrent symbionts in stink bugs [21, 25]. The consistently high prevalence observed in our study strongly suggests that the *Pantoea* strain associated with *P. prasina* has an essential role for the host. Similarly, *Pantoea* spp. have been reported from related pentatomid species including GSB [25]. This role may involve supplying essential amino acids or vitamins that are limited in plant-based diets [63]. *Pantoea* may also assist in detoxifying plant defensive compounds, particularly phenolics abundant in hazelnut tissues [64]. These metabolic contributions could enhance host development and reproductive performance, suggesting that this association may play an important role in the biology of *P. prasina* [21, 25].

Phylogenetic analysis showed that the *Pantoea* strain associated with *P. prasina* clusters within insect-associated lineages. These phylogenetic patterns suggest that insect-associated *Pantoea* strains were acquired repeatedly from environmental ancestors rather than through strict co-diversification with Pentatomidae. Insect-associated *Pantoea* lineages have also been identified in several Pentatomidae species, reinforcing their recurrent association with stink bugs [15, 21, 25, 28]. Although some *Pantoea* species are recognized plant pathogens, the strain associated with *P. prasina* clusters with insect-associated symbionts rather than phytopathogenic lineages. This pattern implies a specialized adaptation to the insect host. Its precise functional impact on *P. prasina* biology and, indirectly, on hazelnut physiology and nut quality remains to be determined.

Experimental removal of symbionts in other pentatomid species, such as *Halyomorpha halys* and *Acrosternum heegeri*, has been shown to increase mortality and delay development, supporting the idea that these bacteria are essential for nutrient provisioning and host fitness [15, 28]. However, a similar effect was not observed in GSB, as reported by [25]. In GSB, treatment with the anti-symbiont biocomplex did not significantly affect first-instar mortality or the presence of *Pantoea* symbionts, which has been attributed to limited symbiont acquisition by the nymphs and the low efficacy of the treatment. These findings indicate that alternative anti-symbiont compounds are needed for use in symbiont removal assays, and that future studies including functional genomic analyses will be necessary to clarify the specific contributions of this symbiont to host performance and feeding on hazelnut.

In addition to *Pantoea*, our results show that *Sodalis* functions as a secondary (facultative) symbiont in *P. prasina*, consistent with patterns reported in other Pentatomidae [19–21]. *Sodalis* lineages are known to be widespread across Hemiptera, although their functional roles vary widely among hosts [21, 65, 66]. In our phylogenetic analysis, *Sodalis* sequences from *P. prasina* clustered into a distinct, well-supported clade, indicating that these strains are evolutionarily differentiated from *Sodalis* lineages described in other stink bugs [20].The substantial variation in infection prevalence among provinces further suggests that *Sodalis* may not be essential for host survival but could influence context-dependent traits such as stress tolerance or nutrient balance. Such geographic differences may reflect microclimatic variation among hazelnut-growing regions, which could alter symbiont acquisition, maintenance, or transmission efficiency. Alternatively, interactions with local plant chemistry or soil-derived microbial communities may differentially favor *Sodalis* persistence across provinces. Similar facultative roles have been reported in other hemipterans, where *Sodalis*-related bacteria can supplement amino acids or modify host physiological pathways [65, 67]. Collectively, these findings indicate that *Sodalis* contributes to the symbiotic landscape of *P. prasina* but likely plays a more flexible and environmentally sensitive role than the obligate symbiont *Pantoea*.

Several methodological limitations should be considered when interpreting these findings. The 16 S rRNA marker provides limited taxonomic resolution for distinguishing closely related *Enterobacteriaceae*, potentially obscuring strain-level diversity, while pooling individuals for sequencing can mask inter-individual variability and underestimate rare taxa. Moreover, DNA was extracted from whole insects rather than dissected gut tissues, preventing exclusive attribution of all detected taxa to the gut; notably, some genera, including *Raoultella* and *Serratia*, have previously been isolated from hemolymph [57], indicating that not all associated bacteria are necessarily gut symbionts. Nevertheless, the dominance and high prevalence of *Pantoea*-like symbionts in our dataset al.ign with gut-focused studies, including [25], which localized primary symbionts to the V4 region of the *P. prasina* midgut. These findings suggest that, while caution is warranted when interpreting whole-insect DNA data, our results capture the main gut-associated symbiotic signal and provide a foundation for understanding host-microbe interactions in this species.

Future research should prioritize experimental validation of symbiont function, particularly through symbiont removal assays and controlled re-inoculation experiments that directly test the physiological roles of *Pantoea* and *Sodalis*. Strain-level comparative genomics will also be essential to identify metabolic pathways underpinning obligate versus facultative associations. In addition, longitudinal sampling across seasons and plant phenological stages could reveal how microbiome dynamics respond to changes in host diet and environmental conditions. Finally, integrating symbiont biology with hazelnut orchard management will be essential for translating microbiome insights into practical, symbiont-based control strategies. This integration will require studies that examine how symbionts influence feeding damage, nut quality, and pest population dynamics.

In conclusion, this study provides the first comprehensive analysis of the symbiotic bacterial community of *P. prasina*, a major pest of hazelnut orchards in Türkiye. Across five key hazelnut-producing provinces, *Pantoea* and *Sodalis* emerged as the predominant symbionts. *Pantoea* infected all examined individuals, whereas *Sodalis* showed variable prevalence among locations. These patterns, together with phylogenetic evidence, support a primary (obligate) role for *Pantoea* and a secondary (facultative) role for *Sodalis*. A deeper understanding of these symbiotic associations will inform the development of symbiont-based strategies that can contribute to sustainable integrated pest management in hazelnut orchards.

## Supplementary Information

Below is the link to the electronic supplementary material.

Supplementary Material 1

Supplementary Material 2

## Data Availability

The datasets generated and/or analyzed during the current study are not publicly available but are available from the corresponding author upon reasonable request.
