# Supplementary material for: Diversity and Composition of the Microbiome Associated with Adult of the Green Shield Bug Palomena prasina (Hemiptera: Pentatomidae)
Source: Microb Ecol. 2026 Apr 29;89:130. doi: 10.1007/s00248-026-02779-2 (PMC13275768; doi:10.1007/s00248-026-02779-2)
Supplement: Supplementary file 1 [file 248_2026_2779_MOESM2_ESM.docx]

**Supplementary Figure 1** Ventral view of the male and female abdomens of *Palomena prasina* showing the morphological differences between sexes.

**Supplementary Figure 2** Stacked bar plot showing the relative abundance of bacterial taxa associated with *Palomena prasina* at the genus level.

**DÜZCE PROVINCE**

**DAF**

**DAM**

**DÇF**

**DÇM**

**DCF**

**DCM**

**GİRESUN PROVINCE**

**GBF**

**GBM**

**GEF**

**GEM**

**GPF**

**GPM**

**ORDU PROVINCE**

**OGF**

**OGM**

**OUF**

**OUM**

**OÜF**

**OÜM**

**SAKARYA PROVINCE**

**SHF**

**SHM**

**SKF**

**SKM**

**SKOF**

**SKOM**

**SAMSUN PROVINCE**

**SOF**

**SOM**

**SÇF**

**SÇM**

**STF**

**STM**
