# Supplementary material for: Diversity and Composition of the Microbiome Associated with Adult of the Green Shield Bug Palomena prasina (Hemiptera: Pentatomidae)
Source: Microb Ecol. 2026 Apr 29;89(1):130. doi: 10.1007/s00248-026-02779-2 (PMC13275768; doi:10.1007/s00248-026-02779-2)
Supplement: Supplementary file 2 — Supplementary Material 2 [file 248_2026_2779_MOESM2_ESM.docx]

**Supplementary Figure 1** Ventral view of the male and female abdomens of *Palomena prasina* showing the morphological differences between sexes.

*
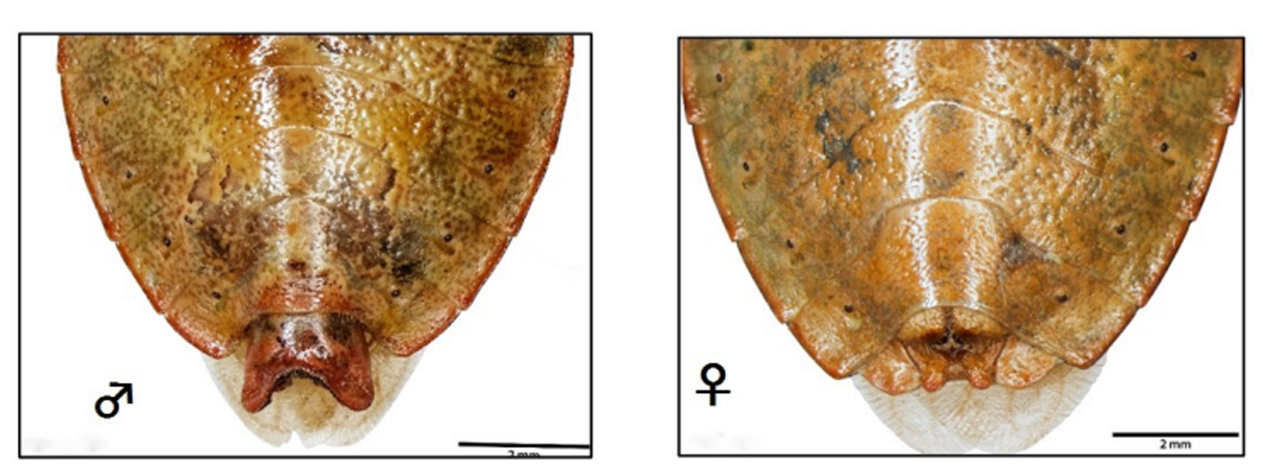
*

**Supplementary Figure 2** Stacked bar plot showing the relative abundance of bacterial taxa associated with *Palomena prasina* at the genus level.

**DÜZCE PROVINCE**

**DAF**

**
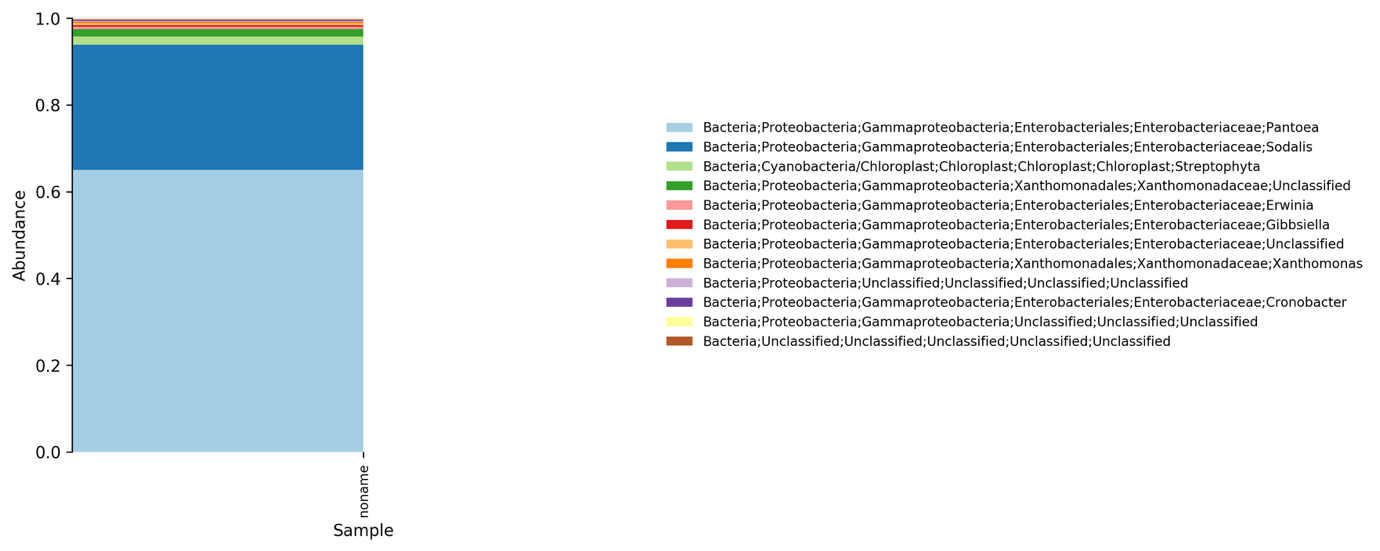
**

**DAM**

**
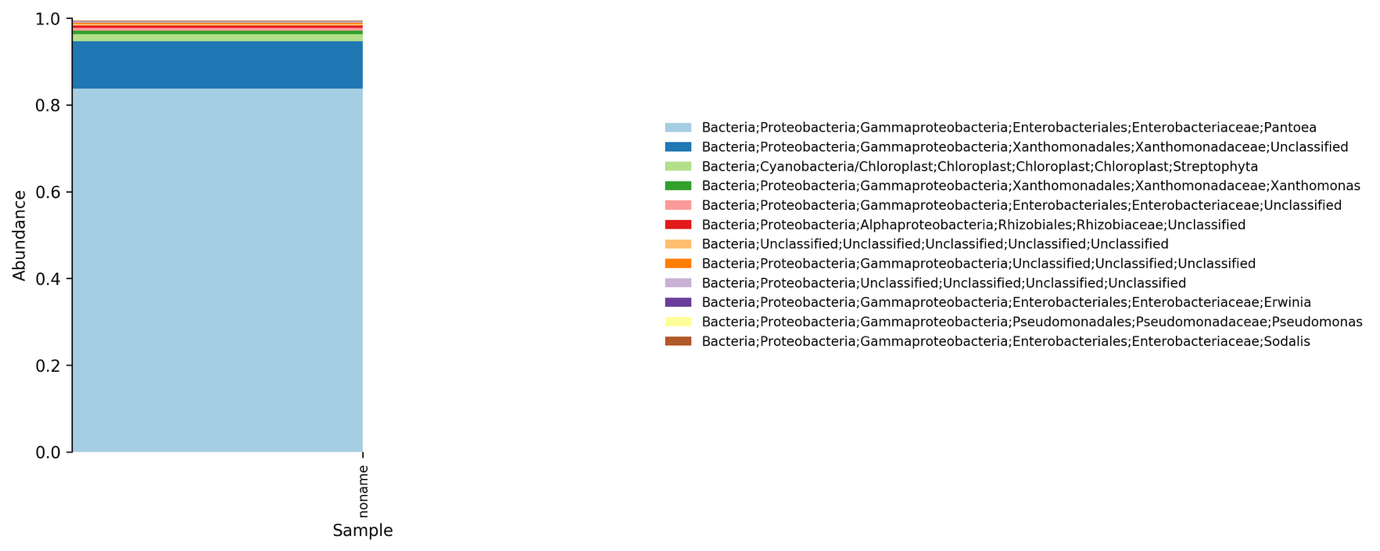
**

**DÇF**

**
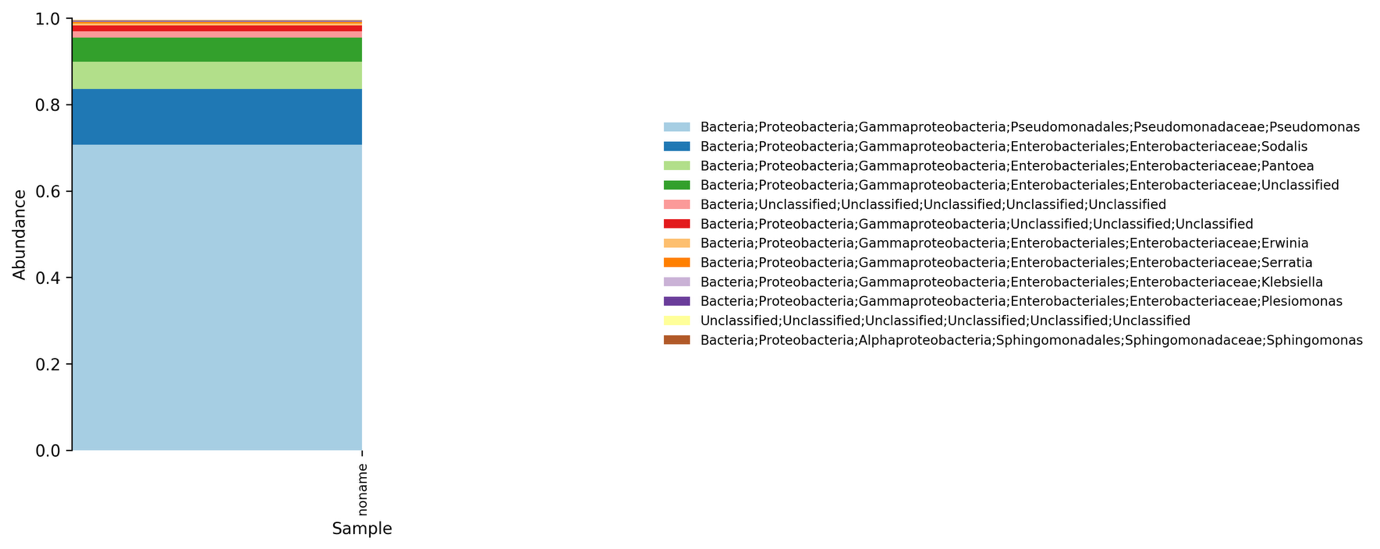
**

**DÇM**

**
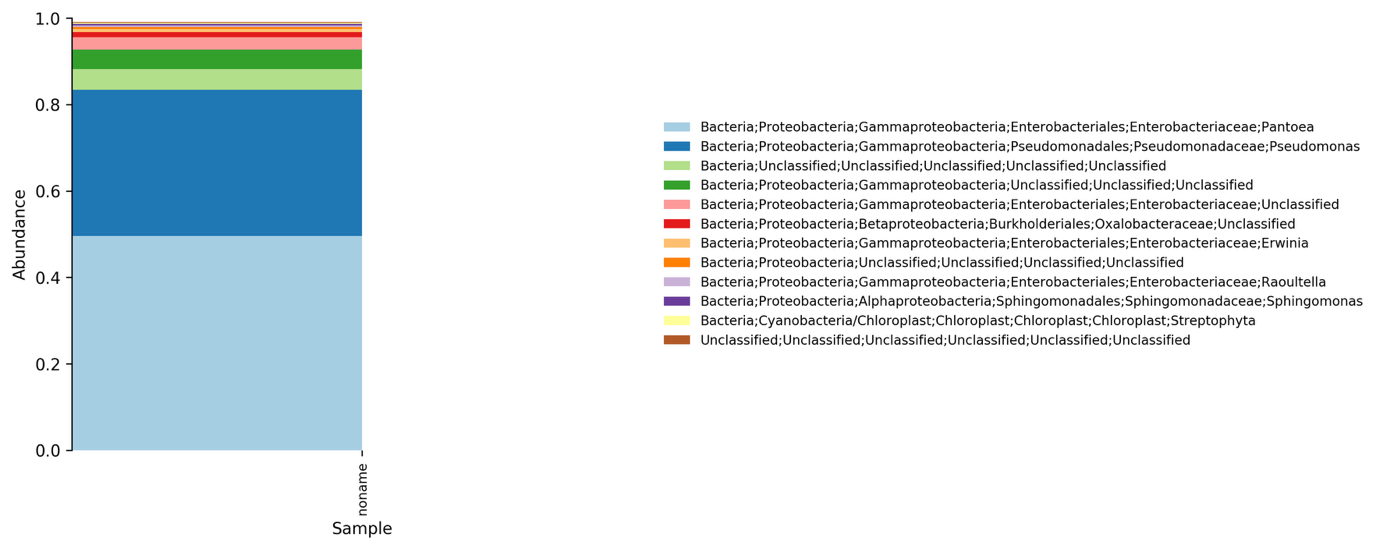
**

**DCF**

**
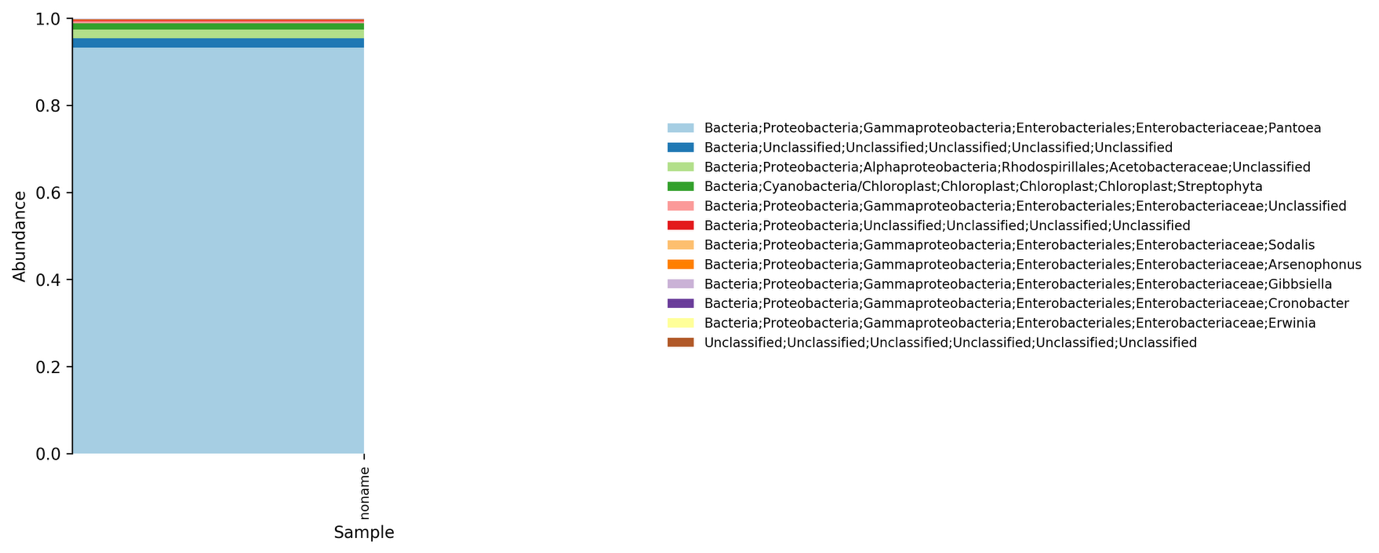
**

**DCM**

**
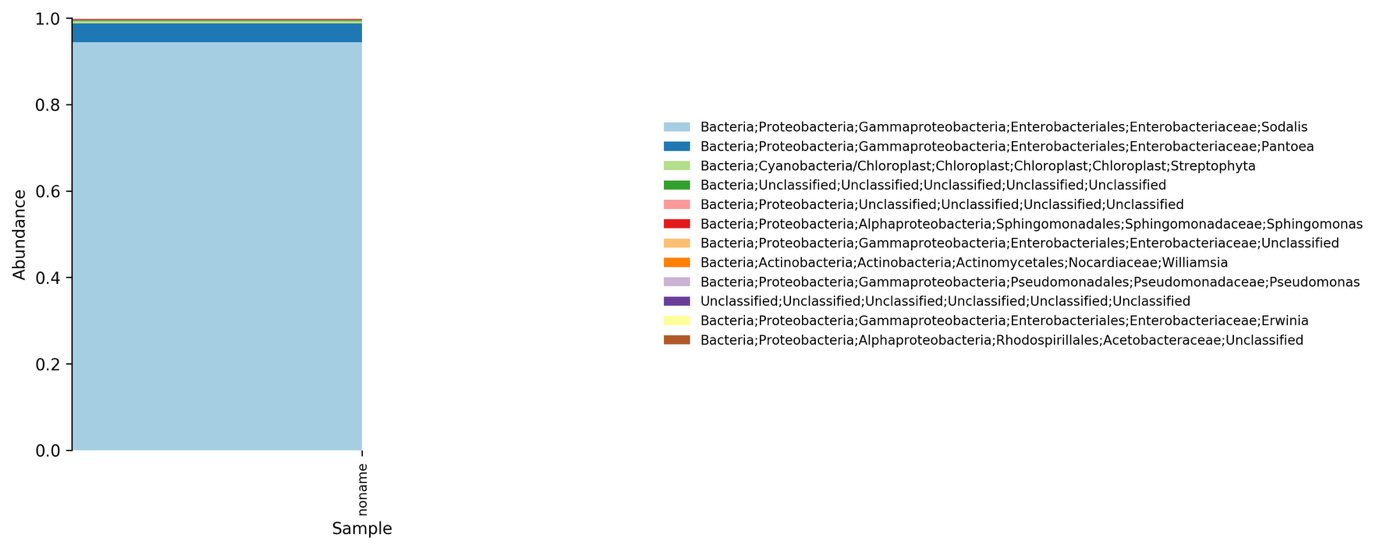
**

**GİRESUN PROVINCE**

**GBF
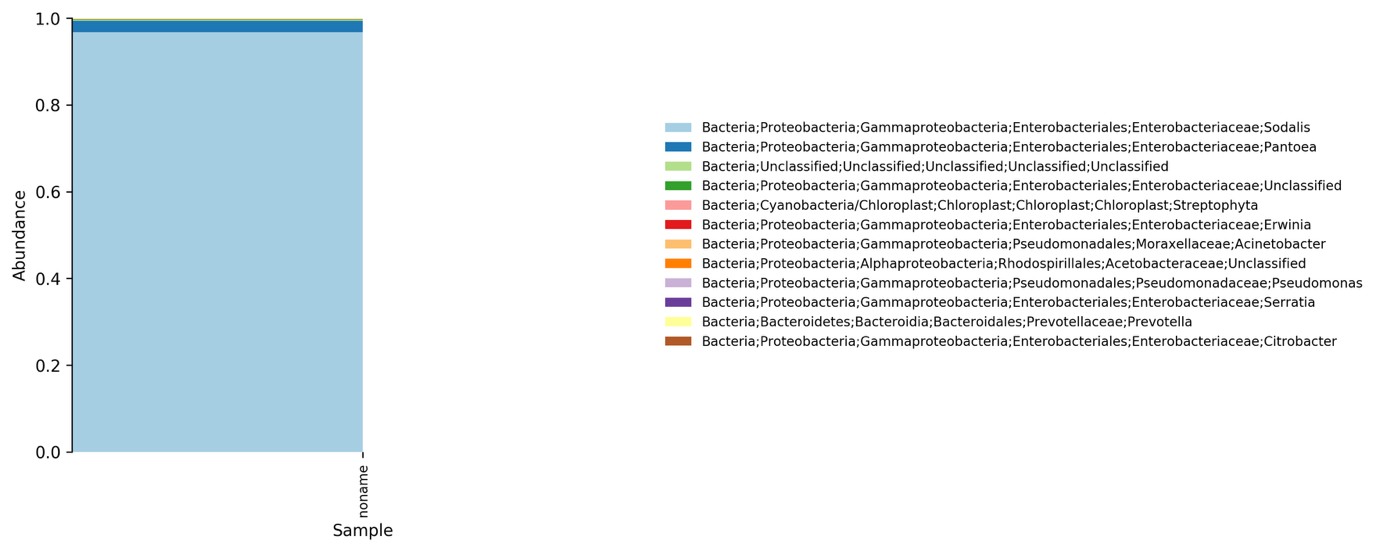
**

**GBM
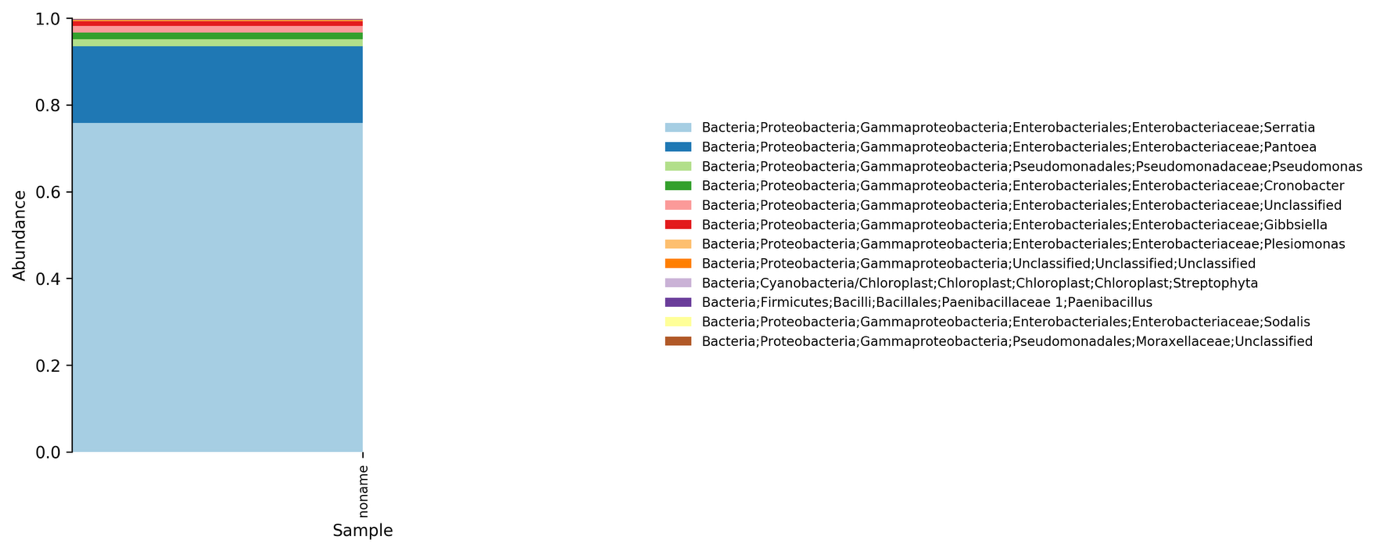
**

**GEF
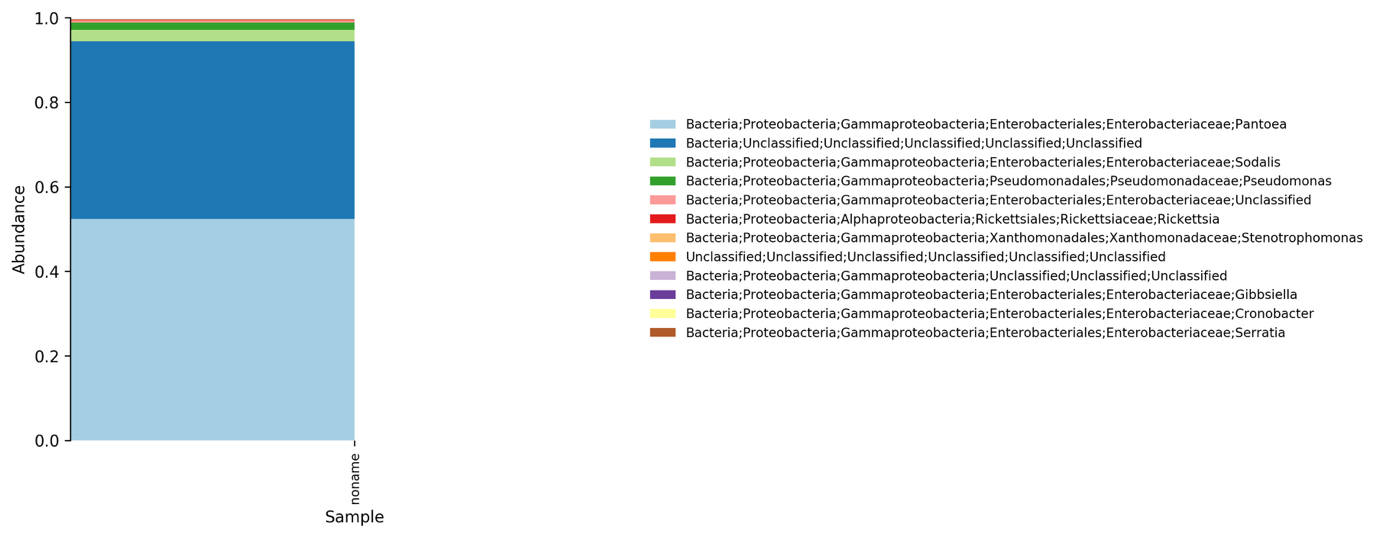
**

**GEM**

**
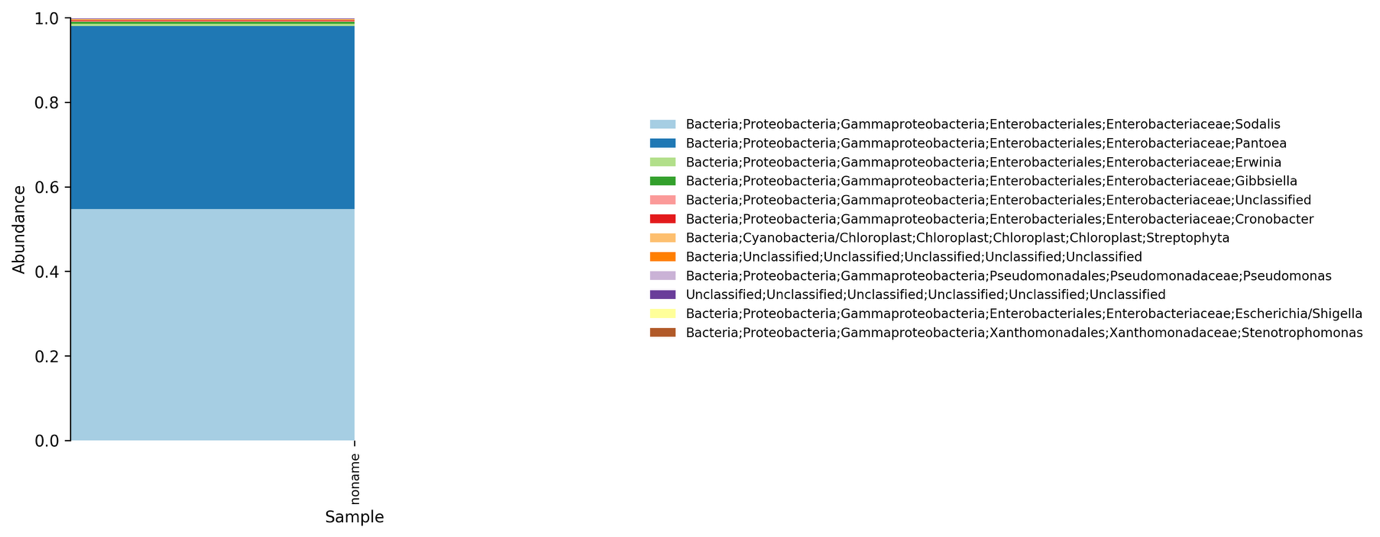
**

**GPF**


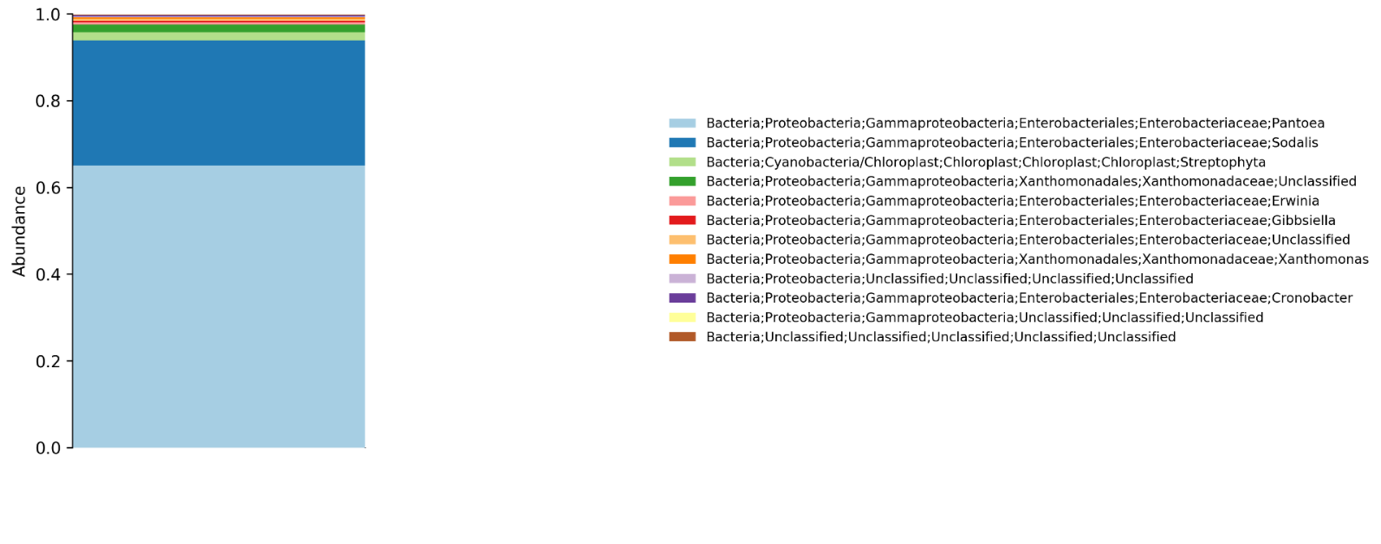


**GPM**

**
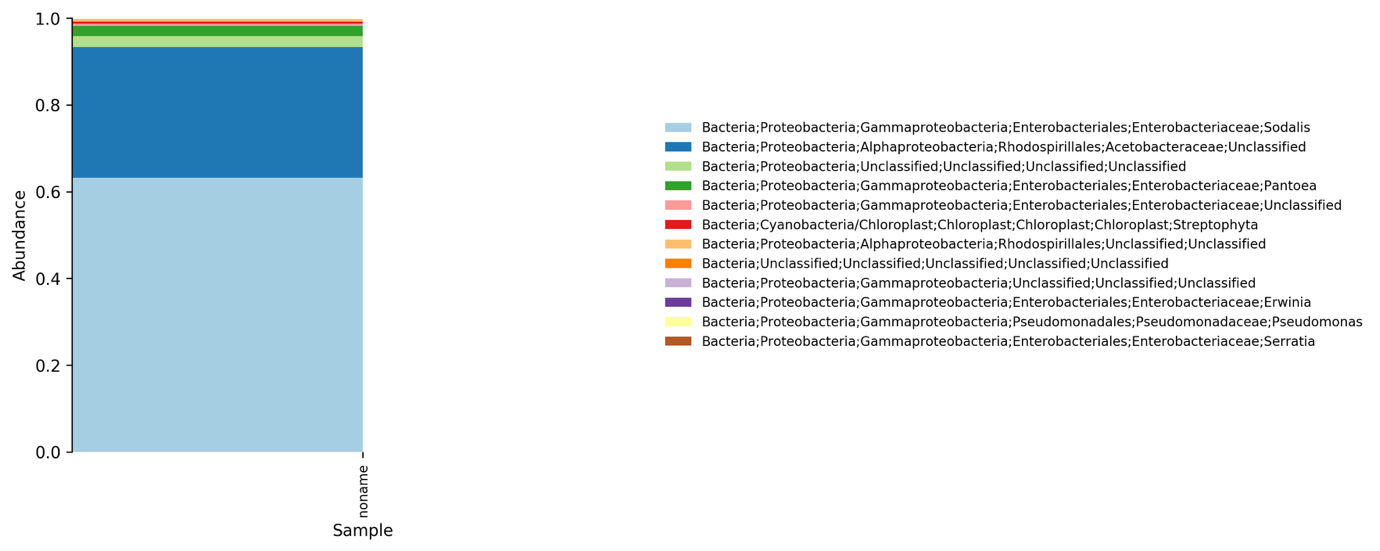
**

**ORDU PROVINCE**

**OGF**

**
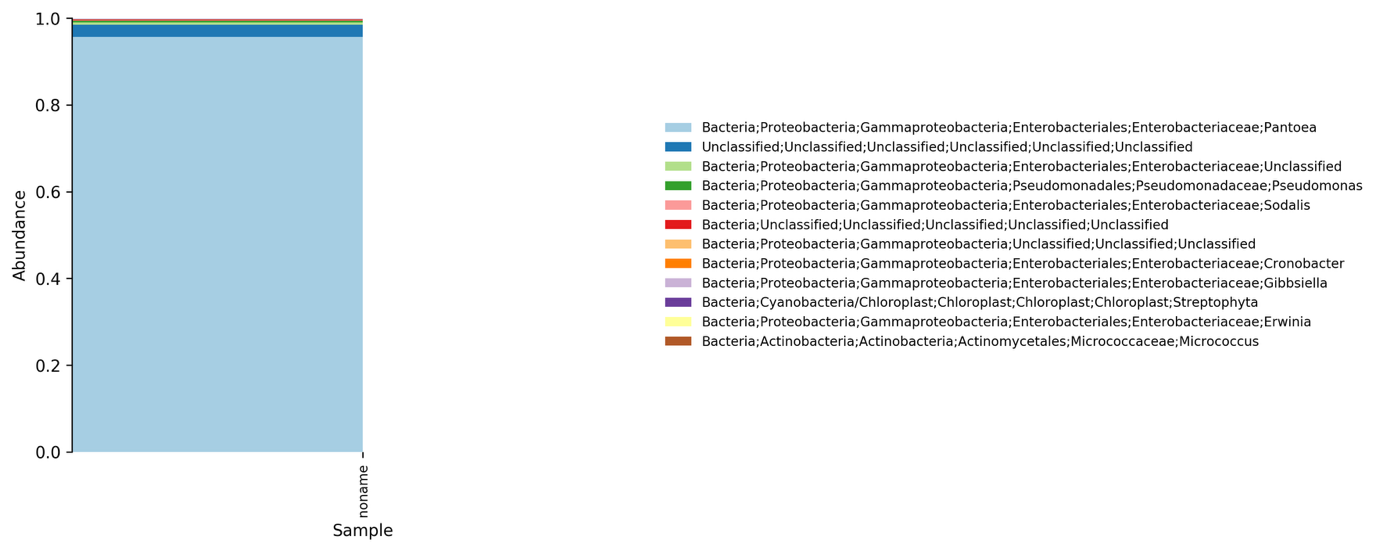
**

**OGM**

**
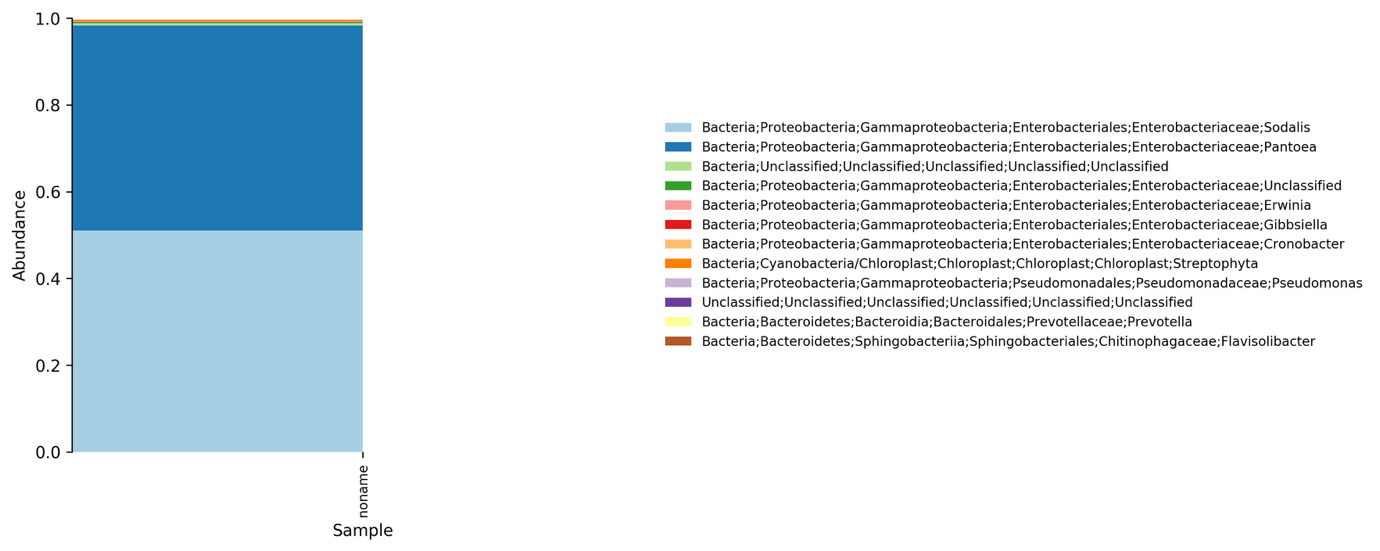
**

**OUF**

**
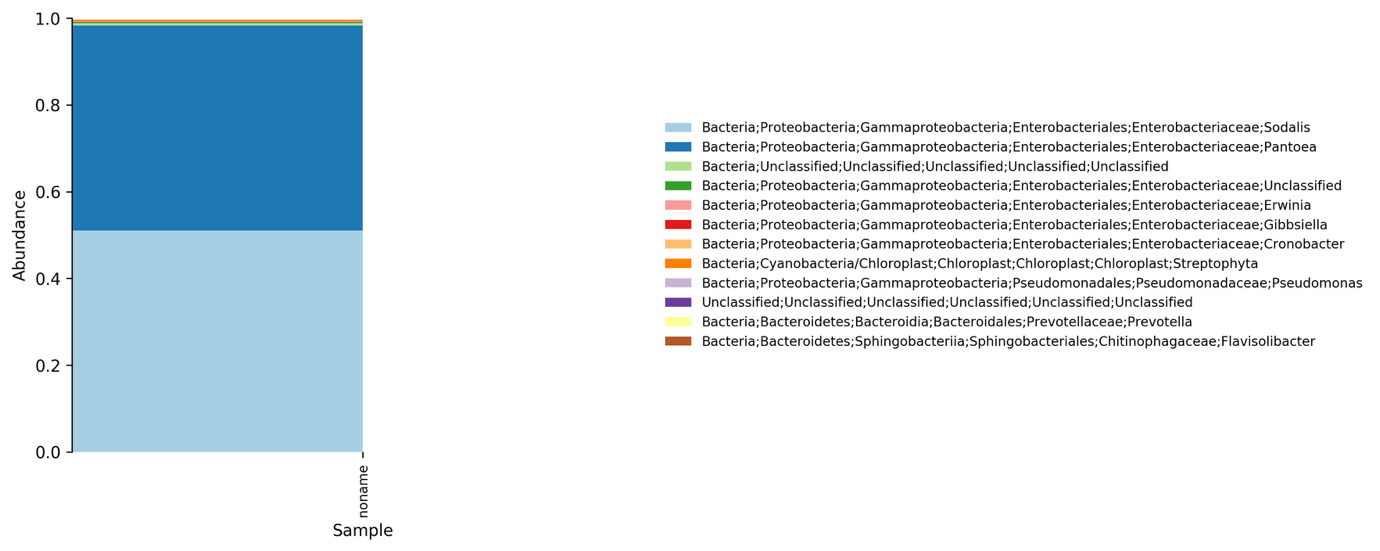
**

**OUM**

**
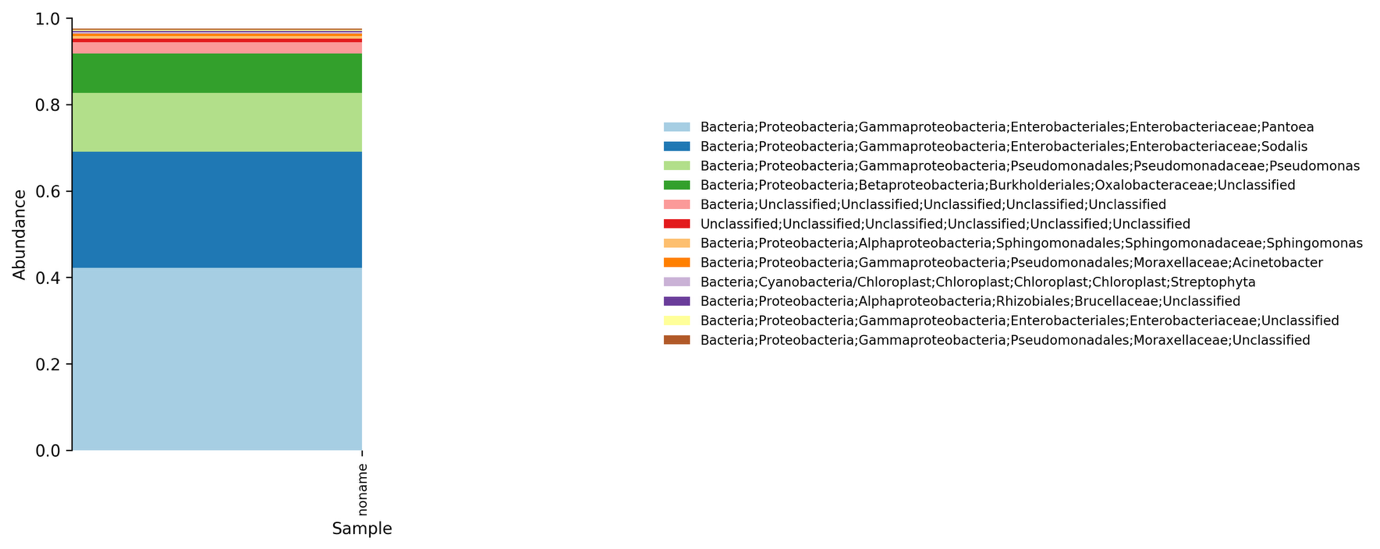
**

**OÜF**

**
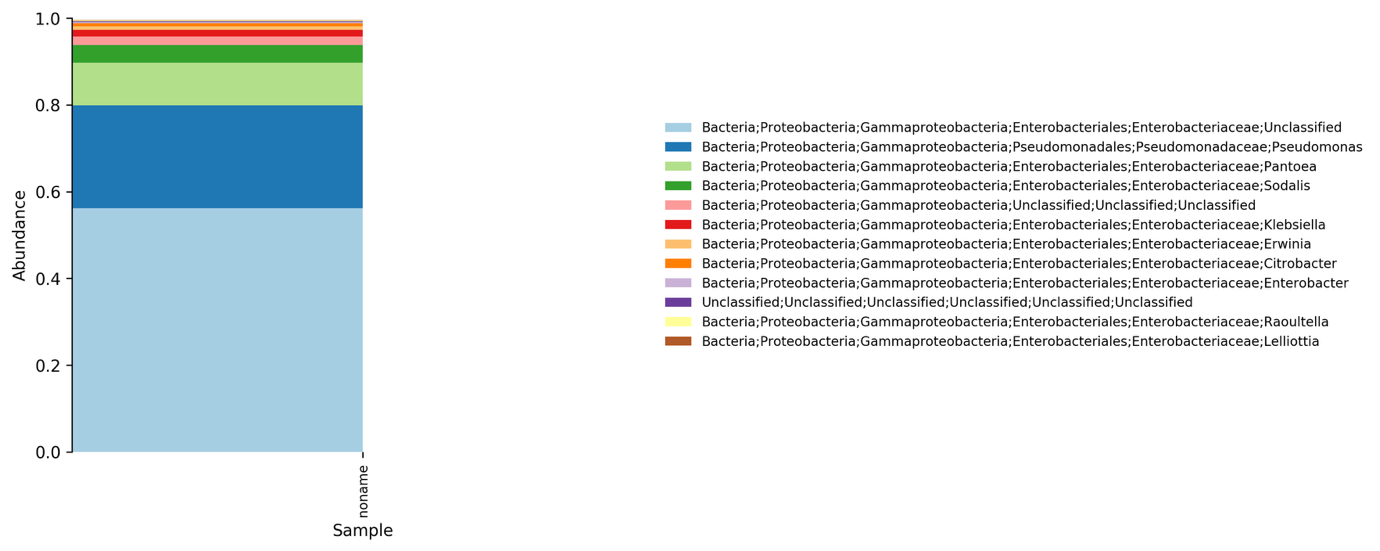
**

**OÜM**

**
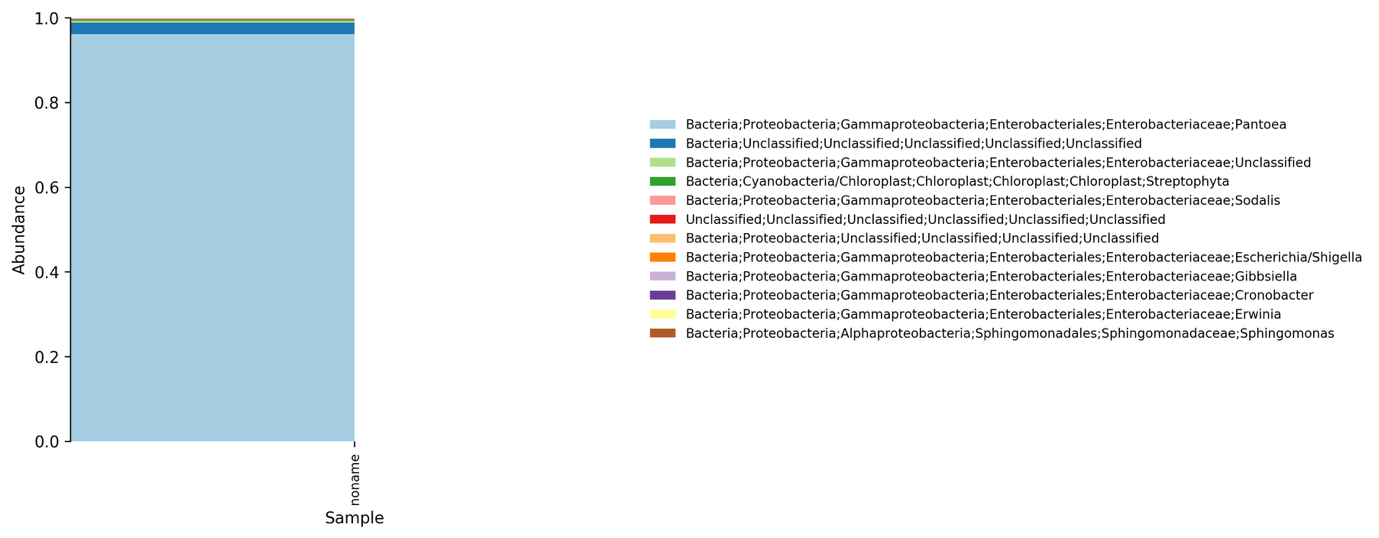
**

**SAKARYA PROVINCE**

**SHF
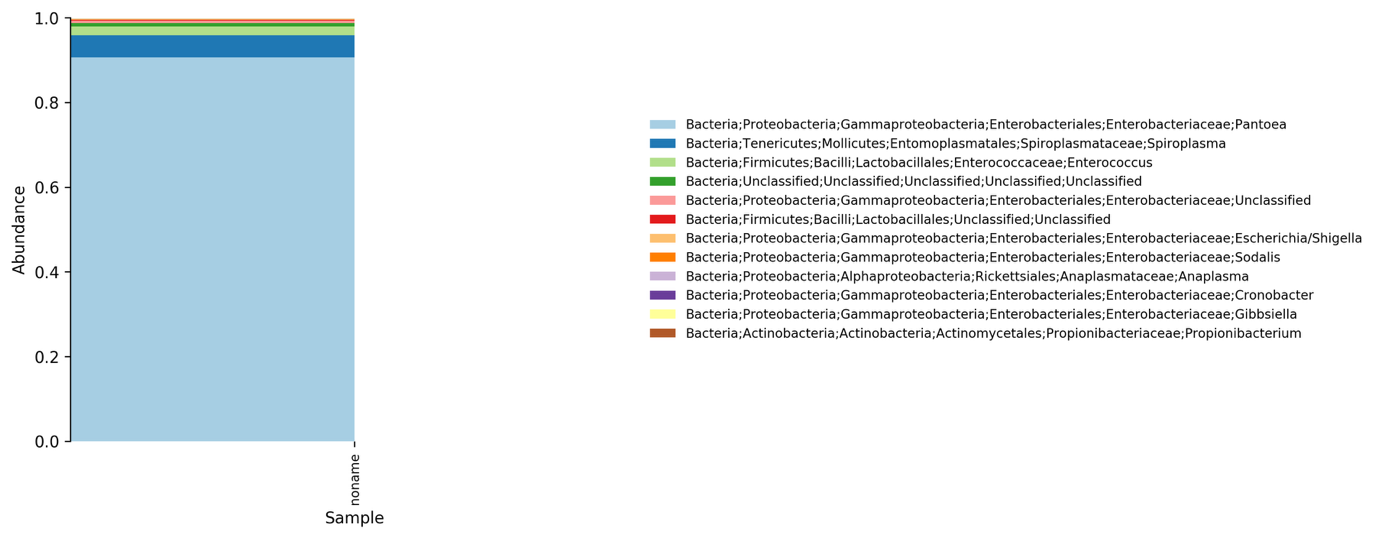
**

**SHM**

**
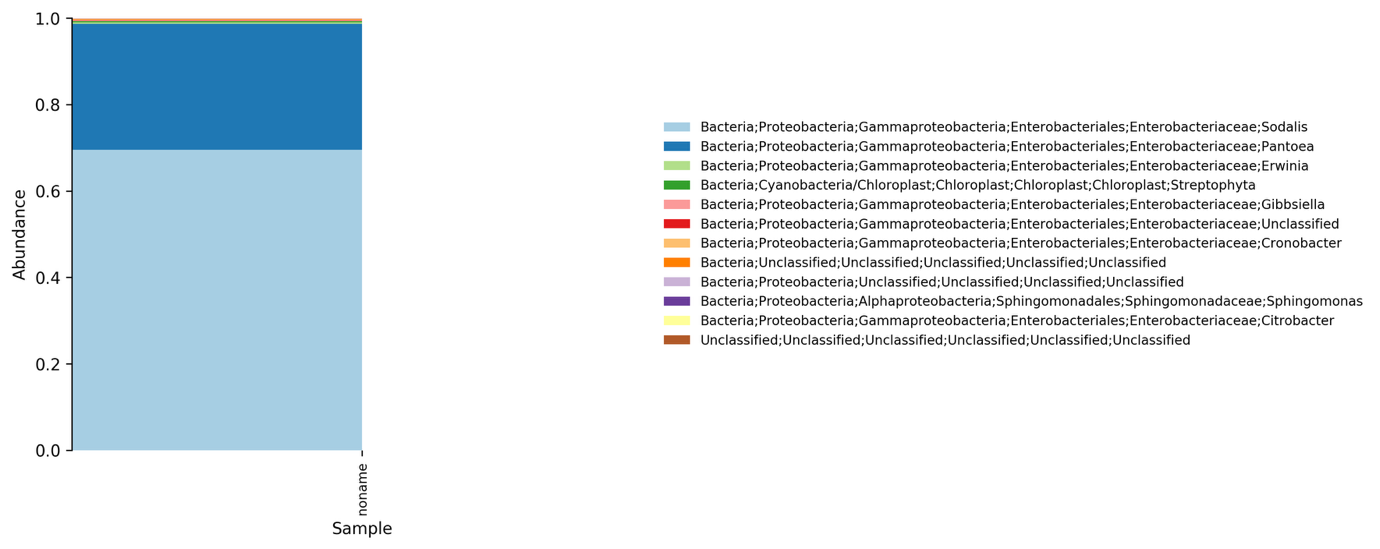
**

**SKF**

**
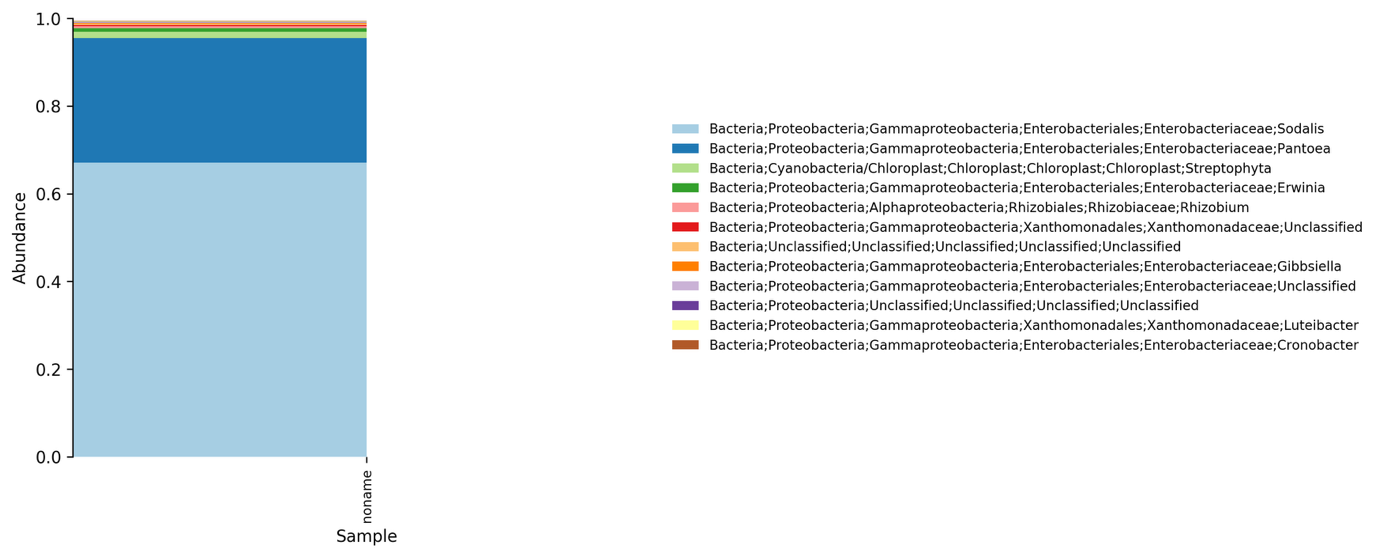
**

**SKM**

**
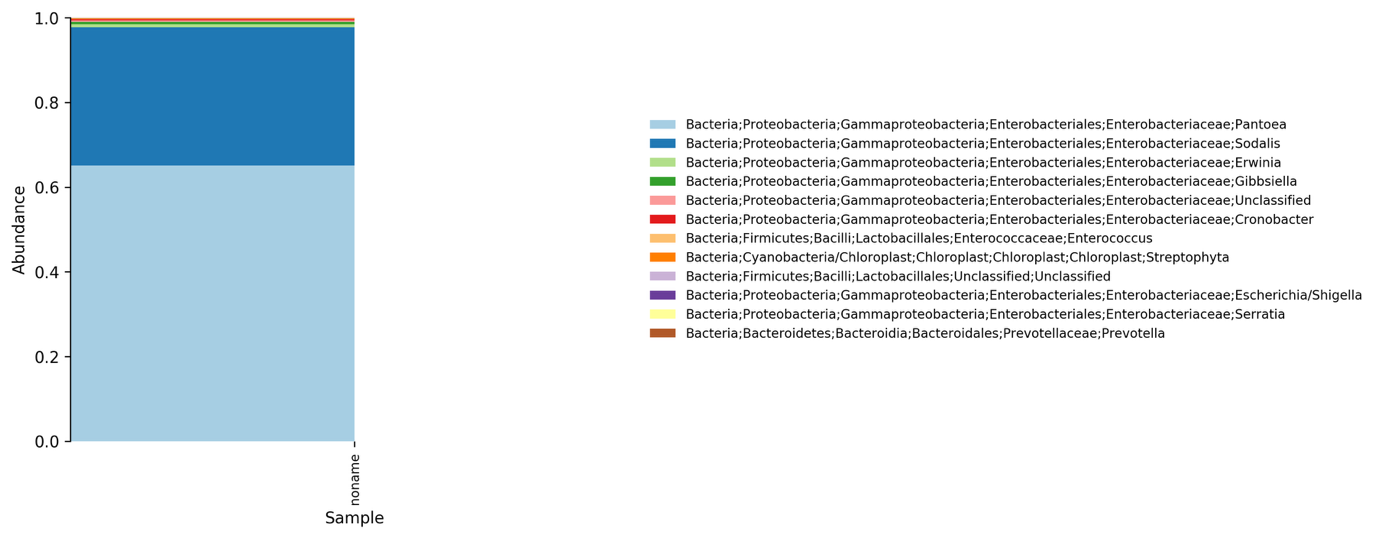
**

**SKOF**

**
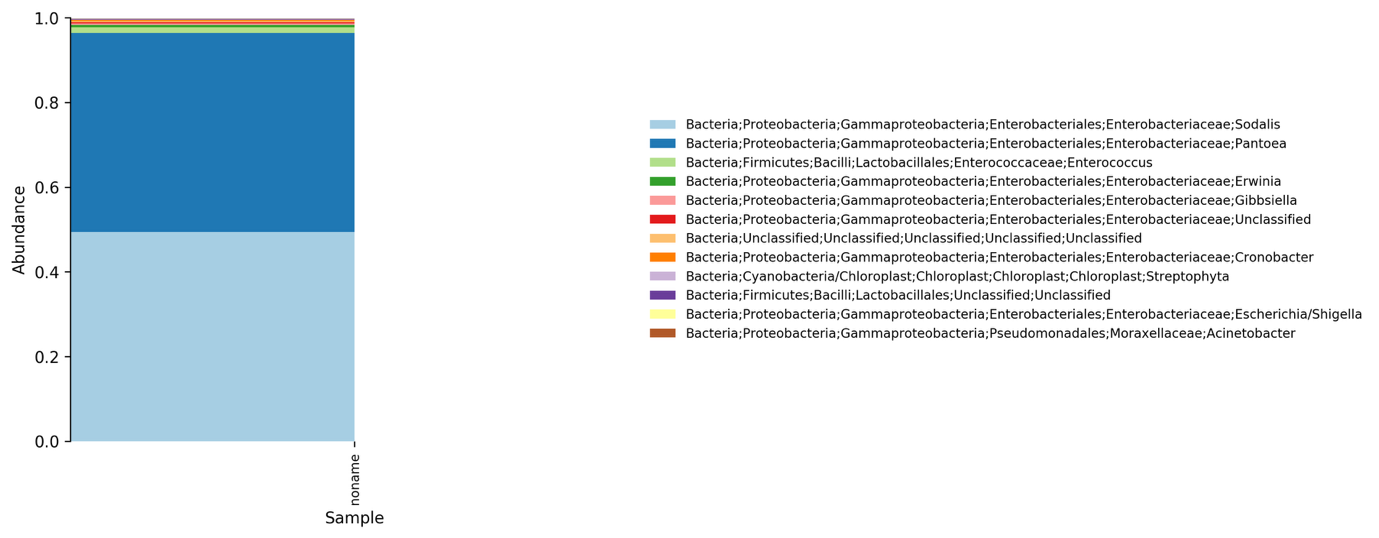
**

**SKOM**

**
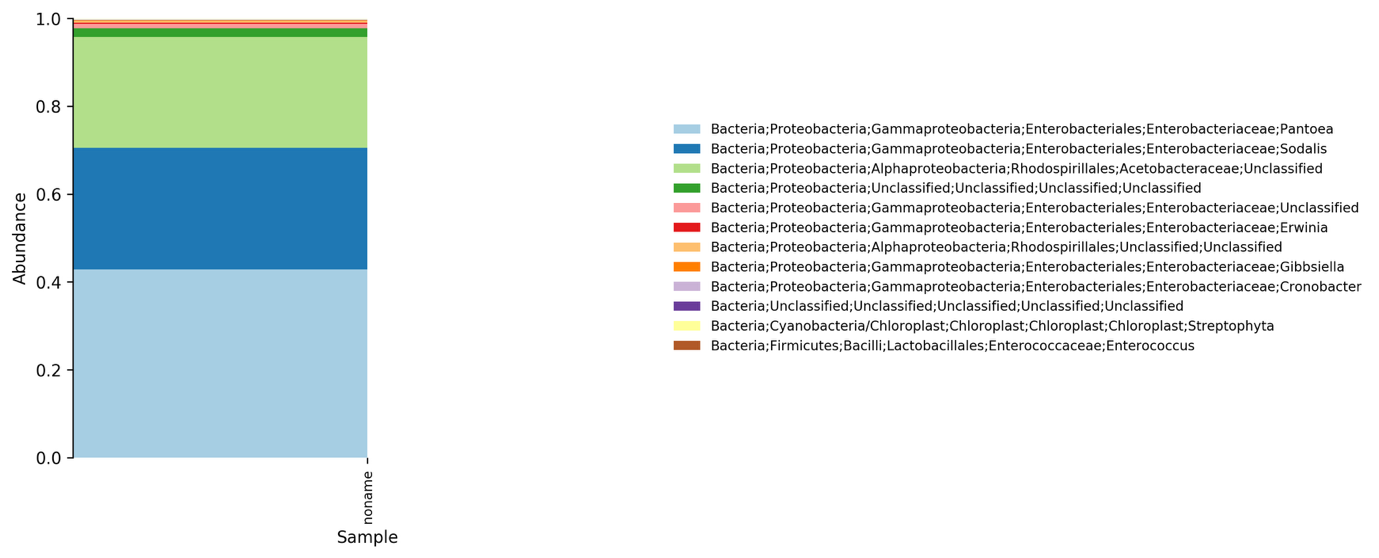
**

**SAMSUN PROVINCE**

**SOF**

**
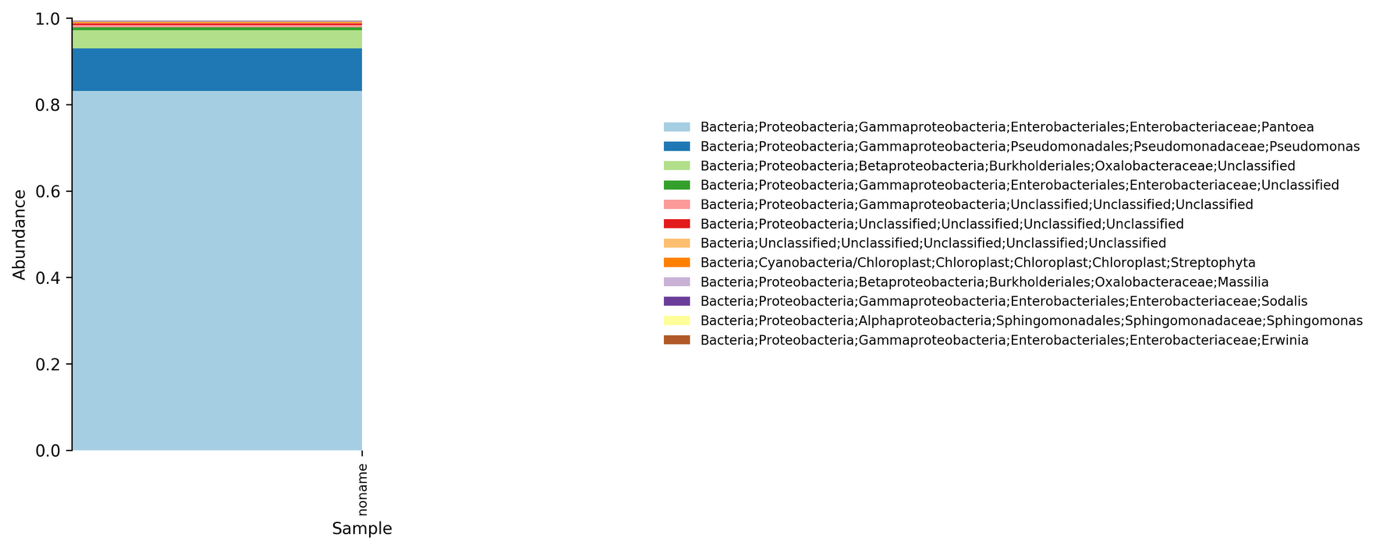
**

**SOM**

**
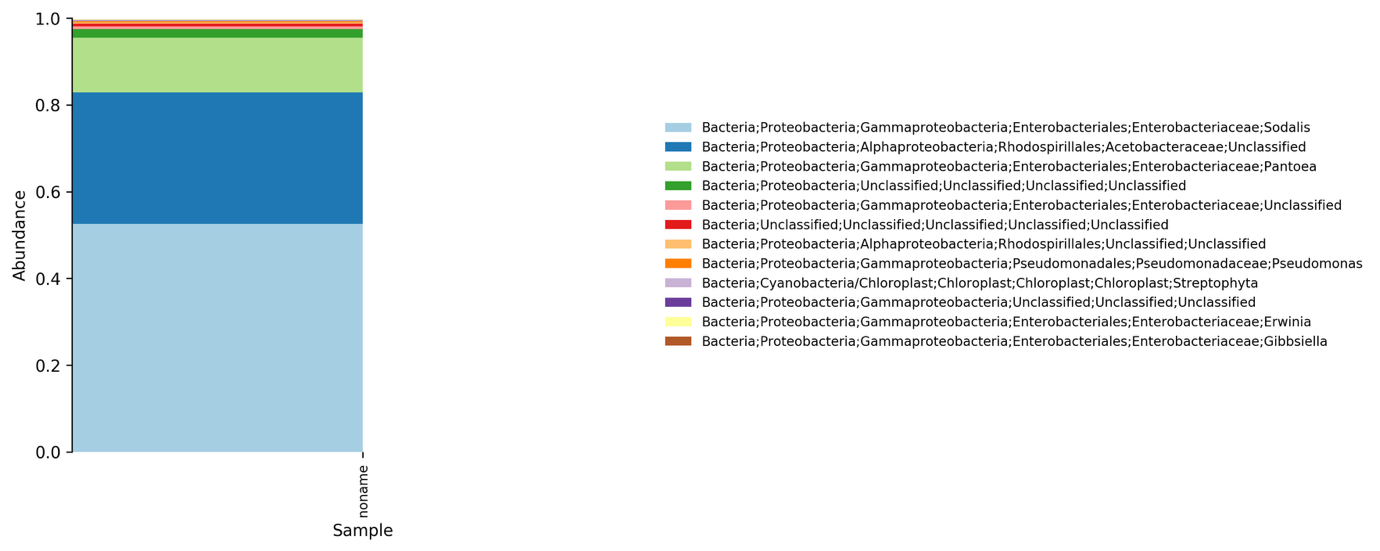
**

**SÇF**

**
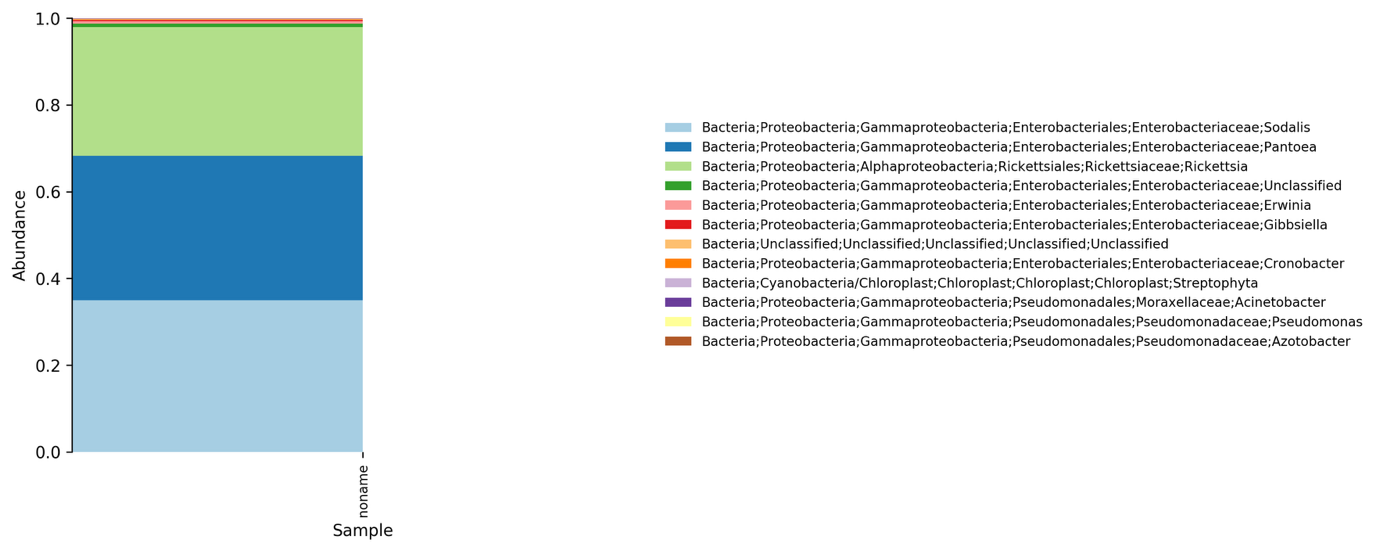
**

**SÇM**

**
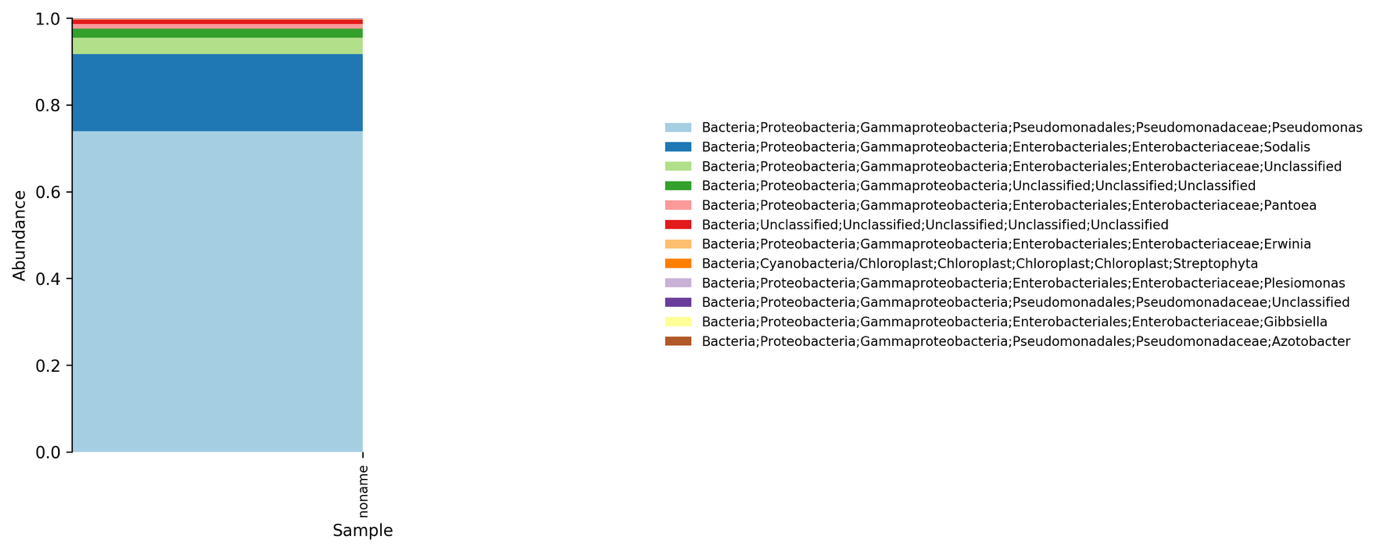
**

**STF**

**
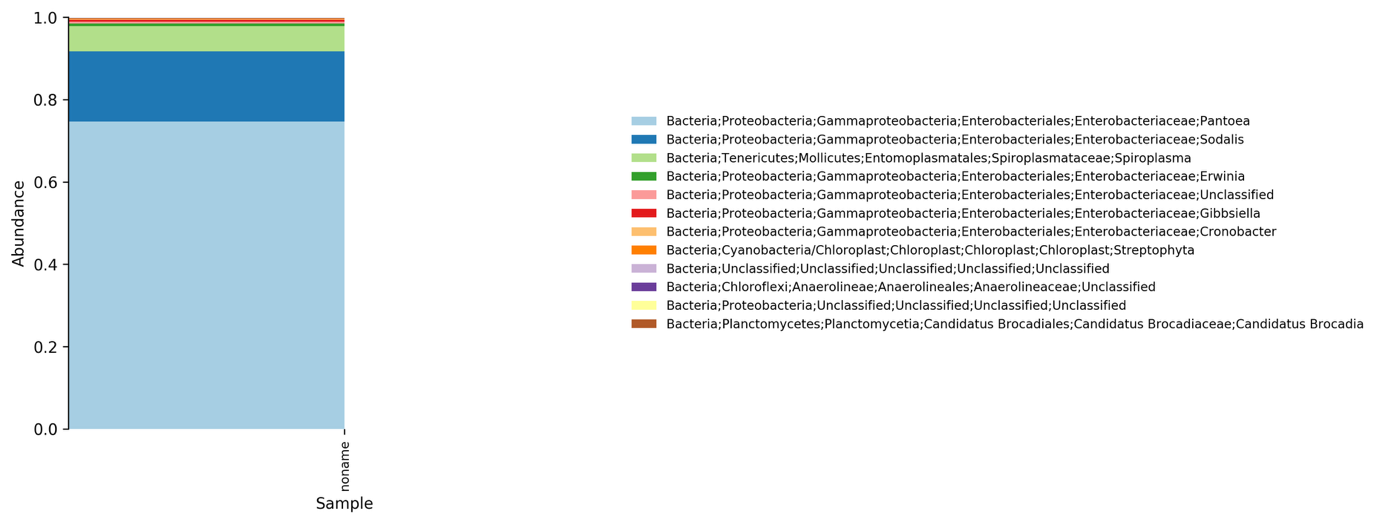
**

**STM**

**
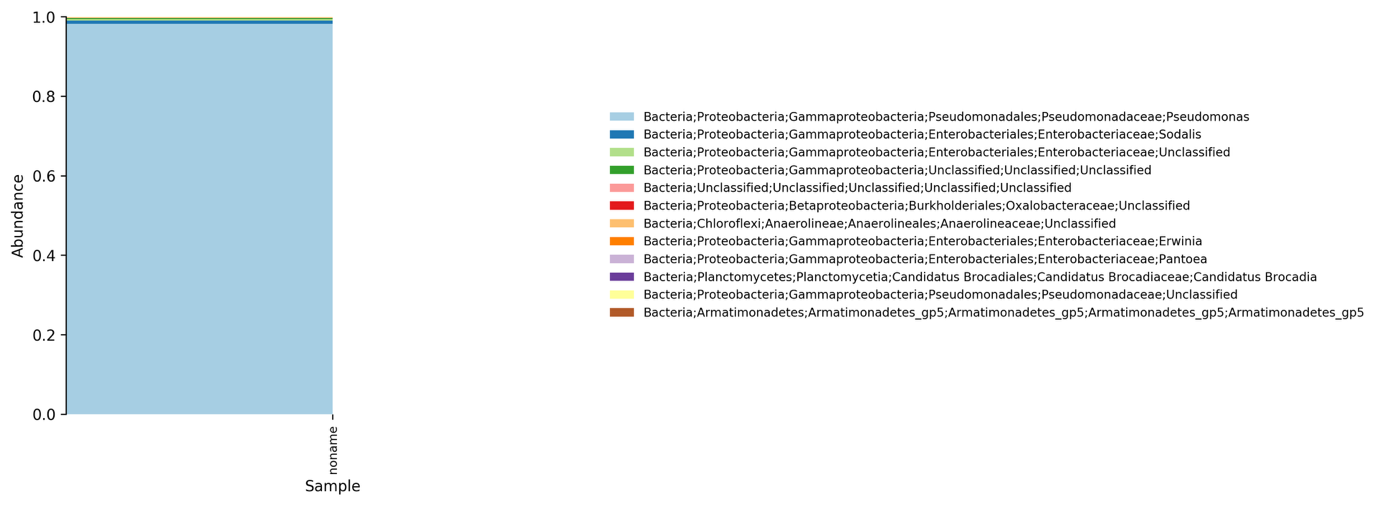
**
